# CO_2_ Electroreduction to Ethylene: The Determining Role of the Cell and Electrode Design

**DOI:** 10.1021/acs.energyfuels.5c02802

**Published:** 2025-09-05

**Authors:** Paolo Squillaci, Georgia Papanikolaou, Siglinda Perathoner, Gabriele Centi, Mattia Melloni, Angelo Ferrando, Paola Lanzafame

**Affiliations:** † Department ChiBioFarAm - Industrial Chemistry, University of Messina, INSTM CASPE (Laboratory of Catalysis for Sustainable Production and Energy) and ERIC aisbl, Viale F. Stagno d’Alcontres 31, Messina 98166, Italy; ‡ Versalis SpA, Basic Chemical & Plastics Research Centre, Via Taliercio 14, 46100 Mantova, Italy

## Abstract

A series of copper-based electrocatalysts, selected from the literature for their high activity in the CO_2_RR to ethylene, is compared under the same conditions, revealing a large discrepancy with the optimal results reported in the literature. A further detailed analysis of a copper oxide nanosheet electrocatalyst under different reaction conditions (cell configuration, i.e., liquid- vs gas-phase, membrane type, i.e., proton exchange membrane (PEM) vs anion exchange membrane (AEM), type of electrolyte, CO_2_ mass transport under flow-field-enhanced conditions, and current density) reveals the dramatic role of cell and electrode configurations in determining selectivity. The Faradaic efficiency (FE) for ethylene production on copper oxide nanosheet electrocatalysts can vary widely, ranging from 0% to nearly 70%, depending on reaction conditions. The discussion of these results highlights how the surface reactivity and conversion pathways critically depend on surface accessibility and the population of CO_2_ and protons, which in turn depend on both surface characteristics beyond the presence of specific active sites and the rate of transport to the electroactive surface. This is a more critical factor in determining selectivity than the mere type of active sites. However, it is noted that this behavior cannot be described by adding mass transfer limitations to the intrinsic electrocatalyst activity, due to the complex interrelation between many aspects beyond the conventional engineering mass transport considerations. This is demonstrated, for example, by operating at high current densities where ethylene selectivity drastically improves. The results also shed light on and provide a different perspective on the literature results and mechanistic-derived conclusions.

## Graphical abstract

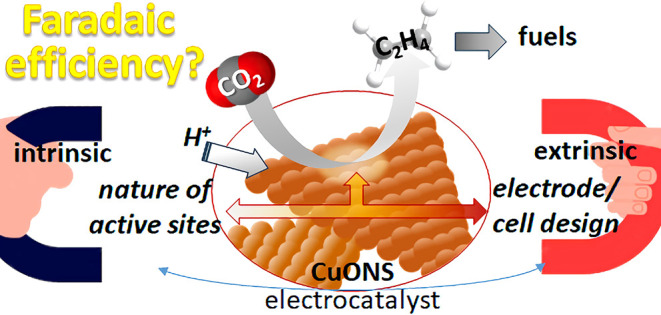

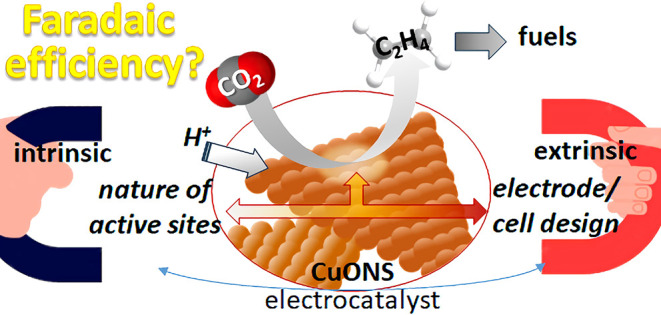

## 1 Introduction

The electrocatalytic reduction of CO_2_ to ethylene is a key objective for producing second-generation electrofuels, which are directly derived from renewable energy sources without relying on intermediate H_2_ gas production.^1^ Avoiding this intermediate step results in process intensification and reduction of energy losses related to forming (and then using) H_2_ molecule intermediates, which create thermodynamic limitations due to the reversibility of the involved reactions, not present when H^+^/e^–^ (in electrocatalytic approach) are used rather than H_2_ molecule (as in thermocatalytic approach).^2^ Thus, the direct production of ethylene from CO_2_ can decrease both operating and fixed costs, potentially meeting the requirements to transition from lab-scale curiosity to application. Ethylene can produce selective jet fuels by selective (catalytic) oligomerization, opening a sustainable pathway to clean, sulfur-free fuels,^3^ which are an alternative to the production (of aviation e-fuels) via methanol or Fischer–Tropsch routes.^4^ These traditional routes produce a broad range of hydrocarbons, which include products of less valuable use, especially considering the possible future of mobility. Instead, the CO_2_ electrocatalytic conversion to ethylene, followed by ethylene oligomerization to jet fuels, offers advantages in terms of sustainability indices. It is thus an attractive route to produce sustainable, renewable jet fuels, with the advantage that ethylene, being a raw material for chemistry, also has alternative markets. In addition, starting from waste CO_2_, it is also an important route for carbon circularity.^5^ The key step in this route is the possibility of producing ethylene competitively by the electrocatalytic reduction of CO_2_.

Numerous studies and reviews have been conducted on the electrocatalytic conversion of CO_2_ to ethylene. Copper-based electrocatalysts are the most commonly used, but the analysis of the literature results does not allow for the unique identification of the preferred one due to the great variety in experimental conditions and types of electrocatalytic reactors, often far from those necessary for industrial scalability.^6–11^

Performance enhancements are typically based on catalyst design considerations informed by reaction mechanism and investigations into the nature of the active sites. These aspects of the electrochemical reduction of CO_2_ to ethylene (and other C_1_ and C_2_ products, such as formic acid, acetic acid, and oxalic acid), as well as the nature of the active sites that enable the formation of C2+ vs C1 products, have been extensively studied, both experimentally and computationally.^12–21^ The general assumption is that the differences observed in electrocatalytic performance are primarily related to the nature of the surface active sites and associated mechanistic implications. The electrode and cell design, as well as the operating conditions, can significantly influence performance in CO_2_ electroreduction (CO_2_RR).^22^ However, this aspect has often been overlooked. Therefore, when discussing catalyst design and related mechanistic features, data are compared under very different testing conditions. On the other hand, it has often not been well clarified whether the observed behavior depends on the intrinsic electrocatalyst factor or other aspects. Another associated general assumption is that the electrocatalytic mechanism can be translated, in general terms, from those for heterogeneous catalysts. For these, mass transport limitations overlap with surface kinetics, i.e., they determine the surface concentration of reactants but do not intrinsically modify the kinetics. Is this assumption of separation between surface kinetics and transport limitations valid also for electrocatalysis? It is clearly a key mechanistic and design question, because if valid, it is correct to maintain the separation of the catalyst design characteristics from those related to avoiding/minimizing mass transport to the electrocatalyst surface. However, if the assumption is not valid, all the considerations regarding catalyst design must be revised. This crucial question is very complex to solve, but, to our knowledge, no attempts have been made in this direction. Thus, a first step in developing a new model for electrocatalyst design is to evidence whether the assumption of kinetic separation is consistent with the experimental data.

This study first aims to compare a selection of copper-based electrocatalysts chosen among those most active in the target reaction and test them under the same conditions and type of electrocatalytic reactor relevant for industrial scalability, i.e., a zero-gap type of flow electrocatalytic reactor with an electrode of sufficient size (about 10 cm^2^) and operation conditions suitable for industrial scalability (in terms of pH and other characteristics of the electrolyte).^22–30^ The aim of this work is not to replicate or validate literature results, as the exact identity of previously reported electrocatalysts cannot be confirmed. Instead, the objective is to systematically evaluate a series of copper-based electrocatalysts, synthesized to closely resemble those reported as highly active for CO_2_ electroreduction to ethylene under consistent and comparable reactor setups and operating conditions.

Then, on a specific catalyst (a copper oxide nanosheet sample) selected among those indicated above, an in-depth study has been conducted on the influence of cell configuration, i.e., liquid- vs gas-phase, membrane type, i.e., PEM vs AEM, type of electrolyte, and CO_2_ mass transport under flow-field-enhanced conditions, as well as current density, etc. The objective here is not to optimize performance through engineering, but to demonstrate how, for the same catalyst, performances vary dramatically, in a range far beyond that which may be expected from the overlap of mass transfer limitations and intrinsic kinetic reactivity. Attention has been put on Faradaic efficiency (FE) as the metric for comparison, because this is the parameter typically associated with the intrinsic reactivity. The discussion focuses on global dependence, rather than on specific parameters varied, because they are components contributing to a unified vision that proves the control of surface accessibility of CO_2_ and protons to electrocatalysts as a conditioning aspect determining the FE and pathways of transformation. Therefore, it is not relevant to discuss the specific aspects and motivations. On the other hand, it is emphasized that understanding this global dependence, rather than analyzing the individual components, is a crucial factor, suggesting that the behavior cannot be described as solely mass transfer limitations overlapping with kinetic surface control. These results reveal novel aspects that impact the way in which electrocatalysts for this demanding reaction (CO_2_ to ethylene) can be designed.

Due to the interlinked dependence between catalyst properties (beyond the mere presence of specific sites) and electrode/cell designs, the objective is thus not an engineering optimization and unifying metrics, but to evidence this interlinked dependence and thus the impact of design and mechanistic considerations. Notably, the data reveal that under industrially relevant conditions, particularly with larger electrode areas than those typically used in the literature, the relative performance of catalysts differs from previously reported trends. It thus contributes to identifying pitfalls and scientific gaps,^23^ as well as providing a different perspective on the abundant literature on the topic and the key factors that must be optimized to accelerate the implementation of CO_2_ electroreduction technologies.

The ultimate goal is to provide evidence and indications that the separation between true kinetic and mass transfer limitations is not valid for these electrocatalysts, and thus, the need to base their design on different principles. While various authors noted the importance of controlling the surface environment of the electrocatalyst or, in general, of reactor design,^29–35^ this aspect is still often considered as additional to electrocatalyst design and the presence of specific sites,^13,16,36–38^ based on the assumption of separation of true kinetics and mass transfer, even if never properly verified.

## 2 Experimental Section

### 2.1 Synthesis of the Electrocatalysts

Table 1 summarizes the electrocatalysts used for the initial comparison, selected from the literature to identify different copper systems and nanostructures active in the CO_2_ electroreduction to ethylene. They were synthesized following the procedure reported in the literature.

**Table 1:** General Characteristics of the Electrocatalysts Synthesised and Tested in CO_2_RR in a Flow Zero-gap Type Cell

Nr.	catalyst	type	synthesis	refs
1	CuONS	copper nanosheet	hydrothermal	^39,40^
2	CuO@C	honeycomb CuO on C	sol–gel	^90^
3	CuAl	Al-doped Cu_2_O	wet chemical reduction	^91^
4	Cu/Cu_2_O	Cu nanospheres	aqueous precipitation method	^92,93^
5	CuNWs	copper nanowires	wet assembling chemical reduction	^94,95^
6	CuMOF	Cu-based catalyst derived from a metal–organic framework	pyrolysis of Basolite C300 (a commercial MOF)	^96,97^
7	Ni-CuNWs	Ni single atom on Cu nanowires	hard template polymerization	^98^

The synthesis of copper oxide nanosheet electrocatalysts (CuONS), which we found to exhibit better performance, is described in more detail below. For other electrocatalysts reported in Table 1, the preparation followed the one described in the indicated references. The details of the synthetic procedure and the morphological, chemical, and structural characterisations are reported in the Supporting Information (SI), where the details of the synthesis procedures and characterization data by SEM, XRD and EDX are reported (Figures S1–S7, where the prefix ’S’ indicates that the data are in the Supporting Information).

#### 2.1.1 CuONS

Cu­(NO_3_)_2_·3H_2_O [Merck, purity ≥99%] and NaOH [Merck, purity ≥ 85%] were used as precursors without further purification. The CuONS electrocatalyst was prepared using the hydrothermal method reported by Ma et al.^39^ First, a blue solution of Cu­(OH)_2_ was prepared by adding 25 mL of 6 M NaOH dropwise to a 25 mL aqueous solution of 0.1 M Cu­(NO_3_)_2_·3H_2_O, under stirring. The resulting solution was kept stirring at 600 rpm for 40 min at room temperature using a magnetic stirrer to obtain a homogeneous solution, which was then transferred to a Teflon-lined autoclave with a maximum volume of 60 mL and capped. The solution was then heated at a rate of 5 °C/min to reach 100 °C and maintained for 12 h. After cooling at room temperature, the catalyst was collected by filtration and washed several times with distilled water until a neutral pH was reached. Finally, the gray product was washed three times with ethanol and then dried at 80 °C overnight in an oven.

### 2.2 Preparation of Working Electrodes (WEs)

Typically, a catalyst ink was obtained by mixing 16 mg of catalyst, 1.6 mL of ethanol and 50 μL of Nafion solution. The ink was then ultrasonically mixed for 30 min to obtain a homogeneous solution. The catalyst ink was then deposited onto the surface (16 cm^2^) of the PTFE-treated (5%) porous gas diffusion layer (GDL) 29BC using a spray coating technique. To ensure rapid evaporation of the ethanol during the entire spraying phase, the GDL was heated to 80 °C using a hot plate. The gas diffusion electrode (GDE) so prepared was then maintained at 80 °C overnight in an oven. The catalyst’s loading (about 1 mg/cm^2^) on the GDE was determined by the difference in weight between the GLD before and after the spray coating.

### 2.3 Electrochemical Cell Configuration

To investigate the influence of reactor design on CO_2_ electroreduction performance, two distinct electrochemical cell configurations were employed: a liquid-flow cell (L-FC) and a gas-flow cell (G-FC). These configurations were selected to enable a systematic comparison between liquid- and gas-phase operation modes under otherwise consistent testing conditions.

The following section provides a detailed description of the cell design and operational configuration used in this study.

#### 2.3.1 L-FC

Figure 1a presents a schematic representation of the cell in the L-FC configuration. The cathodic compartment, located on the left, contains the catholyte, which flows through the chamber. A catalyst layer is coated onto the GDE, which serves as WE. A reference electrode (Ag/AgCl) is positioned in close proximity to the working electrode. An ion-exchange membrane separates the cathodic and anodic compartments. The anodic compartment contains the anolyte, where oxygen evolution occurs, and houses the counter electrode (Ir/Ru dimensionally stable anode DSA plate). In this configuration, CO_2_ and catholyte are introduced from the left side, while the mixture of catholyte and gaseous products exits through the top and bottom left outlets, respectively. Meanwhile, the anolyte enters from the right side and exits with oxygen at the top right outlet.

**Figure 1:**
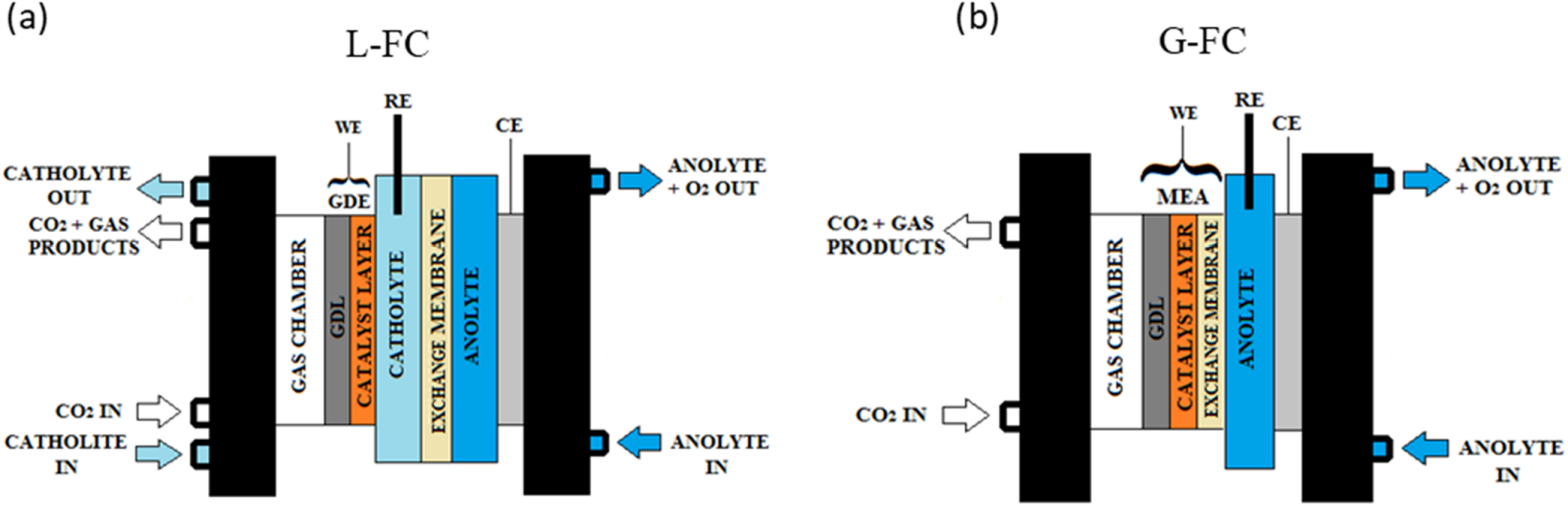
Cell configurations used for CO_2_RR: (a) Liquid/gas phase (L-FC); (b) Gas-phase (G-FC).

#### 2.3.2 G-FC

Figure 1b illustrates the schematic layout of the cell in the G-FC configuration. In this setup, the catholyte is absent, and the WE is fabricated using membrane electrode assembly (MEA) technology by pressing the GDE against an ion-exchange membrane, which also serves as a solid electrolyte. In contrast to the L-FC configuration, where the RE is located in the cathodic compartment, the RE in the G-FC configuration is immersed in the anolyte. The counter electrode, which is also an Ir/Ru DSA plate in this case, is positioned within the anodic compartment. CO_2_ flows from the left, reacts at the MEA, and the resulting gaseous products exit through the bottom outlet. The anolyte flows into the anodic chamber from the bottom right and exits with oxygen from the top right outlet.

For both configurations, the catholyte (if present) and anolyte tanks were externally connected to the FC. An external peristaltic pump was used to facilitate the recycling of the electrolyte and was set at a flow rate of 15 mL/min. Pure CO_2_ was flushed throughout the test at a rate of 10 mL/min using a mass flow controller.

### 2.4 Electrochemical Measurements

The CO_2_RR reaction was carried out in a flow cell assembly (Figure S8 showing the setup of the electrocatalytic cell) purchased from ElectroCell, operating at ambient conditions. The cell is powered by a potentiostat/galvanostat (Autolab PGSTAT302N, Metrohm). The electrocatalytic system operates in a three-electrode cell configuration, utilizing the Cu-based GDE as the working electrode (WE), an Ir/Ru DSA plate as the counter electrode, and a 1 mm leak-free Ag/AgCl (3.5 M KCl) electrode as the reference. The used flow cell is composed of three separate compartments: two liquid chambers (cathodic and anodic) and a gas flow chamber. The liquid compartments were physically separated by two different exchange membranes: Fumasep-FAA-3-PK-75 and Nafion 115, which had been previously purified and activated (described in the SI). Anodic and cathodic electrolytes were contained in two externally separated tanks (40 mL), connected to the cell and circulated at a flow rate of 15 mL/min using a peristaltic pump (Ismatec). During all the electrochemical tests, pure CO_2_ (99.999%) was continuously flushed into the gas chamber (behind the GDE) at a flow rate of 12 cm^3^, as measured by a mass flow meter. CO_2_ was humidified using a bubbler filled with ultrapure water before it flowed into the flow cell. The gas outlet was connected to a digital flow meter to register the outlet flow during the electrocatalytic tests.

Prior to CO_2_ electrocatalytic tests, all the GDEs were studied by electrochemical impedance spectroscopy (EIS) in a frequency range from 100 kHz to 0.1 Hz with an amplitude of 30 mV.

A series of preliminary tests was made to verify that ethylene forms from the reduction of CO_2_ sent through the GDE electrode.

Linear sweep voltammetry (LSV), cyclic voltammetry (CV), and double-layer capacitance (*C*_dl_) measurements of the materials were performed in a three-electrode system (Figure S9, showing a picture of the three-electrode system used for electrochemical characterization). LSV experiments were conducted from 0.84 to 1.04 V (vs RHE) at a scan rate of 5.0 mV/s. *C*_dl_ and the electrochemically active surface area (ECSA) were determined by scanning CV curves at nonfaradaic regions at varying scan rates (100, 50, 20, 10, and 5 mV/s).

The ECSA can be estimated by using the equation:

1 $$\mathrm{ECSA}=C_{\mathrm{dl}}/C_{s}$$

where *C*_s_ is the specific capacitance of a smooth, planar surface of the same material, typically obtained from literature. In the case of Cu materials, the *C*_s_ used was 0.029 mF/cm^2^ as reported in literature.^40^

### 2.5 CO_2_RR Product Analysis

The gas phase was analyzed by an in-line Agilent 490 micro gas chromatograph (Micro GC) calibrated by using a standard mixture of gases before measurements. A Molsieve 5 Å (N_2_, O_2_, H_2_, CH_4_ and CO) and a PoraPLOT Q (CO_2_, C_2_H_4_, C_2_H_6_, C_3_H_6_ and C_3_H_8_) columns were used to separate gaseous products. In contrast, a thermal conductivity detector (TCD) was used to analyze all gaseous products. The analysis was conducted every 20 min. The FE of gas products was calculated as follows:^13^

2 $$FE\;\left(\%\right)=\frac{Q_{gas}}{Q_{tot}}\times 100\%=\frac{\mathrm{Ne}\times F\times \%V\times v\times P}{60s\times J\times R\times T}\times 100\%$$

where Ne is the number of transferred electrons for a specific product (e.g., 12 for CO_2_ reduction to C_2_H_4_), *F* = 96,485 C/mol is the Faraday constant, %*V* is the volume concentration of gas products obtained by a GC, *v* is the outlet gas flow rate measured, *P* = 1.01·10^5^ Pa is the atmospheric pressure, *J* is the steady-state current, *R* = 8.314 J/mol/K is the gas constant, *T* = 298.15 K is the room temperature.

Liquid products (HCOOH, CH_3_COOH, and H_2_C_2_O_4_) were analyzed by ion chromatography (MetrOhm) equipped with an anionic column and two detectors (UV–vis and Conductivity), using calibration curves. FE of liquid products was determined with the following eq 3:

3 $$\mathrm{FE}\;\left(\%\right)=\frac{Q_{\mathrm{liquid}}}{Q_{\mathrm{tot}}}\times 100\%=\frac{\mathrm{Ne}\times F\times c}{J\times t}\times 100\%$$

where Ne is the number of transferred electrons for a specific product (e.g., 2 e^–^ for CO_2_ reduction to HCOOH), *F* = 96,485 C/mol is the Faraday constant, *c* is the moles of liquid product, *J* is the recorded current, and *t* is the reaction time.

## 3 Results and Discussion

### 3.1 Comparison of the Performances of the Cu-Based Electrocatalysts

Figure 2 compares the performances of the Cu-based electrocatalysts indicated in Table 1 with those reported in the literature. The full-circle symbols represent the best values cited in the references listed in Table 1. These data were obtained under different reaction conditions, using different types of reactors, electrodes, and electrode sizes. The open circle symbols represent the performance observed for analogous electrocatalysts synthesized in this study. The data correspond to the highest C_2_H_4_ FE achieved within the −1.6 to −2.0 V potential range (complete data are provided in Table S1 reporting the full data performances of the synthesized electrocatalysts in the G-FC cell setup). All data were obtained using a flow-type zero-gap reactor configuration, in which the cathodic compartment operates without a liquid electrolyte. Hence, it is briefly referred to as the gas phase. CO_2_ is fed through a gas diffusion electrode (GDE), with the electrocatalyst (at a loading of 1 mg/cm^2^) positioned between the GDE and the Nafion membrane. CO is the main product formed during the reactions (see Table S1). The electrodes used have an active surface area of about 10 cm^2^, which is larger than that employed in several of the cited studies. As noted in the introduction, the objective is not to validate the literature results but rather to compare them under consistent conditions using a flow gas-phase electrocatalytic testing setup.

**Figure 2:**
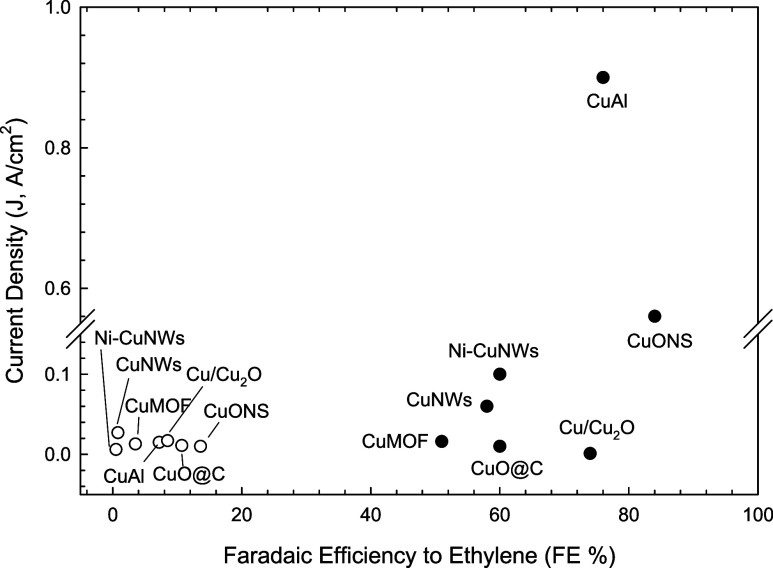
Comparison of the best performances (FE vs J) reported in the literature for the selected electrocatalysts indicated in Table 1 (full circle symbols) with those we obtained for analogously prepared electrocatalysts (open circle symbols). See text for further indications.

A significant discrepancy exists between the performance data reported in the literature and those obtained in this study. One might reasonably question whether these differences arise from imperfect replication of the catalyst structures. To address this, we have selected samples that yield the best FE in our conditions (CuONS and CuO@C) and tested them under different reactor designs, electrode configurations, and operational conditions to determine whether their performances can be improved to become comparable to those reported in the literature. Some further details on the characteristics of the two selected electrocatalysts are presented in Figures S1 and S2, respectively. These supplementary figures report the morphological, chemical and structural characterization of CuONS and CuO@C electrocatalysts. Even accounting for potential differences due to the independent reproduction of the electrocatalysts, the variation in performance is remarkably large. Moreover, even the relative performance ranking of the electrocatalysts differs from that reported in the literature. Therefore, the reactor/electrode design and the operative conditions have a drastic influence that masks the specific characteristics of the electrocatalyst.

To support this point, we performed a comparative analysis of ECSA-normalized metrics for our most active catalyst, CuONS, and a corresponding reference material from the literature.^40^ The results are summarized in Table 2 and Figure S10. The latter reports CV curves and electrochemical double-layer capacitance results. Both ECSA values were calculated using the same specific capacitance value reported in the literature to ensure consistency in the normalization procedure. As shown, although the geometric ECSA values are comparable (34.5 cm^2^ vs 27.9 cm^2^), the normalized C_2_H_4_ FE and the ECSA-normalized current density differ significantly. The literature sample exhibits a normalized C_2_H_4_ FE of 2.32% and a normalized current density of 1.74 mA/cm^2^, whereas our sample achieves only 0.49% and 0.36 mA/cm^2^, respectively. These discrepancies clearly indicate that intrinsic catalyst surface area alone cannot account for the observed performance variations. Instead, they highlight the critical influence of external parameters such as cell configuration, local CO_2_ availability, pH gradient, and interfacial electric field. This comparison reinforces our hypothesis that electrocatalytic activity and selectivity must be interpreted not only in terms of catalyst composition and structure but also within the context of the entire electrochemical system.

**Table 2:** Comparison of ECSA Normalized Faradaic Efficiency to Ethylene and Current Density

electrocatlysts	ECSA (cm^2^)	normalized C_2_H_4_ FE (%)	normalized J (mA/cm^2^)
CuONS^40^	34.5	2.32	1.74
CuONS^a^	27.9	0.49	0.36

a Data derived from this study.

To further demonstrate the above indications, the following sections focus on a single electrocatalyst (copper oxide NS), investigating the role of various electrode/cell design aspects to analyze how these aspects influence the catalytic behavior. We used C_2_H_4_ FE as the unifying metric to compare the results, because this metric is most typically associated with the intrinsic surface behavior of the electrocatalyst. As commented in the introduction, the aim here is not to optimize, from an engineering perspective, the electrode/cell, nor to specifically analyze and discuss the influence of each of the many parameters investigated. Rather, the objective is to assess from a broad perspective how all these parameters are part of the overall picture, showing that (i) the variations in C_2_H_4_ FE are far beyond the variations which may be expected from the simple presence of mass transfer limitations overlapping to the true kinetic behavior, (ii) there are many interlinked aspects which show how the separation between true kinetic and mass transfer is not correct in these electrocatalysts, and (iii) choice of the electrode/cell characteristics as well as of the testing conditions may affect so largely C_2_H_4_ FE to question whether discussions on electrocatalyst design are reliable. Demonstrating these aspects is the first indication that a different approach to designing electrocatalysts is necessary.

### 3.2 The Role of Cell Configuration

Studying electrocatalyst activity for the CO_2_RR in the gas phase is essential due to several key advantages. Direct contact between gaseous CO_2_ and the catalyst surface overcomes the solubility limitations of CO_2_ in aqueous electrolytes, thereby enhancing the local CO_2_ concentration and improving both efficiency and selectivity toward C_2_+ products.^41,42^ Gas-phase configurations also support higher current densities,^43,44^ which are critical for industrial scalability, and typically involve simpler, more cost-effective cell designs. Additionally, gas-phase operation can influence reaction pathways, offering greater control over product distribution. Despite these benefits, most CO_2_RR studies in the literature are conducted in the liquid phase, which presents inherent limitations. These include low CO_2_ solubility (∼33 mM), restricted mass transport, and thicker electrolyte layers that increase diffusion distances. Furthermore, gas bubble formation can block active sites and compromise the electrocatalyst’s stability, while the accumulation of intermediates may alter local conditions and affect selectivity. In between these situations, there are also intermediate situations where the electrocatalyst is in the presence of a thin film of liquid electrolyte. These conditions are those in which salt precipitation may occur,^45^ but on the other hand, they allow for improved conductivity and thus reach higher current densities.

Few studies have compared these different configurations; however, we have observed that they significantly influence the FE and the type of products formed.^22,46,47^ Figure 3 compares the performance of CuONS and CuO@C electrocatalysts under the two cell L-FC and G-FC configurations. All experimental parameters were held constant to ensure a reliable comparison, isolating the influence of the cell architecture on CO_2_ reduction performance. A comparative evaluation of FE toward ethylene as a function of applied potential reveals that the cell configuration has a significant impact on ethylene production for both catalyst compositions. The G-FC configuration consistently yields higher C_2_H_4_ FE across the investigated potential range, highlighting the critical role of gas-phase operation in promoting C–C coupling pathways. This enhancement can be attributed to several interrelated factors. In the L-FC setup, direct contact between the catalyst and the liquid electrolyte results in a higher proton availability at the electrode surface, which favors the competitive hydrogen evolution reaction (HER).

**Figure 3:**
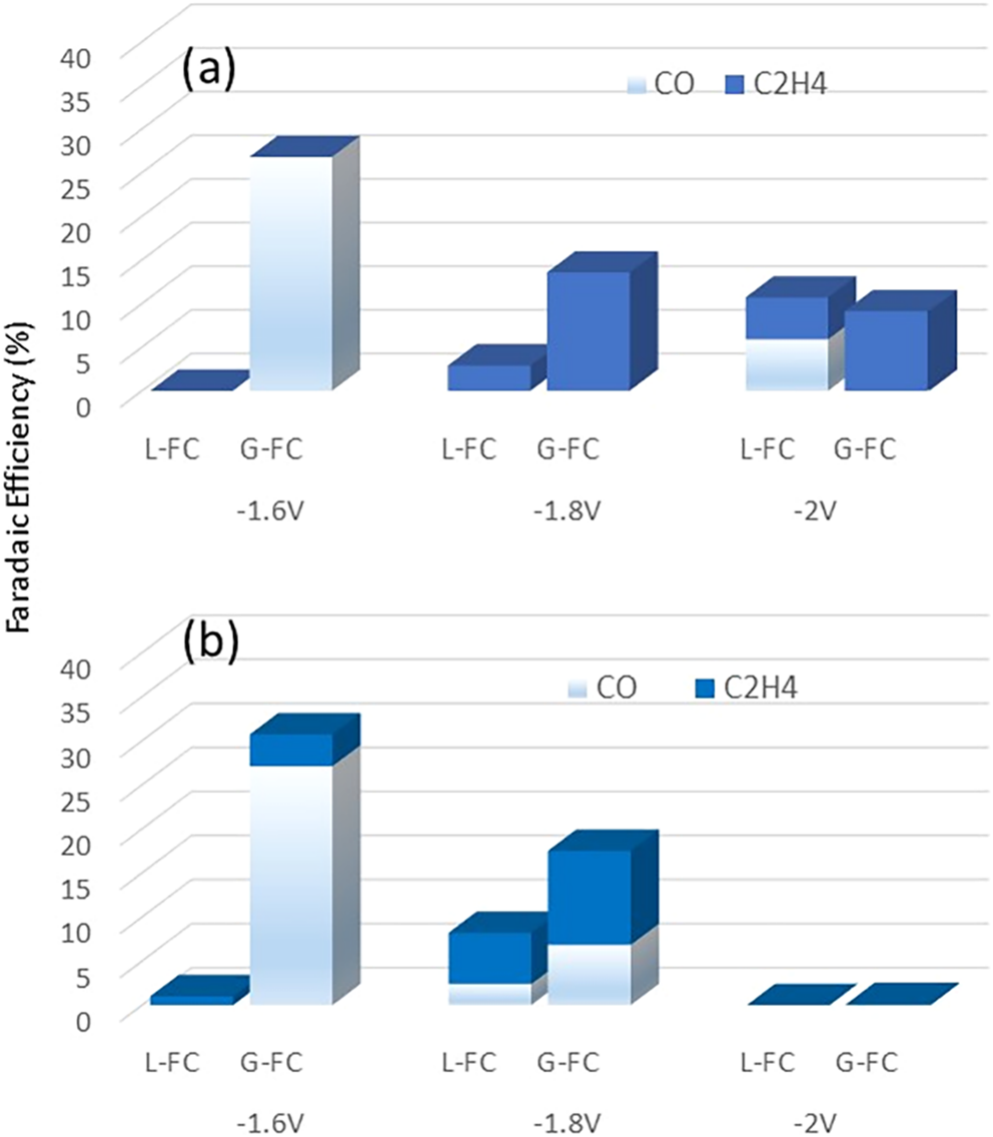
Comparison of the performances (as a function of the applied potential) for the CuONS (a) and CuO@C (b) electrocatalysts in the two cell configurations L-FC and G-FC. *Reaction conditions*: CO_2_ flow: 12 mL/min, electrolyte flows: 15 mL/min, L-FC: 0.1 M KHCO_3,_ both anolyte and catholyte. G-FC 1 M KHCO_3_ anolyte).

The dominance of HER in this configuration reduces the surface coverage of CO_2_ and key intermediates (e.g., *CO), limiting the formation of multicarbon products such as ethylene. Moreover, the presence of a bulk electrolyte likely increases the local dielectric constant and stabilizes proton-involved transition states, thereby further promoting HER over CO_2_RR. Conversely, the G-FC configuration, where the catalyst is interfaced with gaseous CO_2_ and only indirectly exposed to the electrolyte via the membrane, reduces local proton activity at the catalyst surface.^7,48,49^ This shift in interfacial environment serves two beneficial functions for CO_2_RR:

(i) It suppresses HER by limiting proton availability, thereby allowing more electrons to participate in CO_2_ reduction;
(ii) It enhances *CO surface coverage, which facilitates the rate-determining C–C coupling step crucial for ethylene formation. This scenario aligns with previous mechanistic studies suggesting that a moderate *CO coverage is necessary for efficient dimerization and further hydrogenation to ethylene.

Notably, the trend of decreasing CO production with increasingly negative potentials, accompanied by a corresponding rise in ethylene formation (up to −1.8 V), supports a sequential reaction mechanism in which CO acts as an intermediate in the formation of ethylene. However, at −2.0 V, ethylene FE drops again, suggesting a resurgence of HER dominance. This fact is likely due to the overaccumulation of electrons and increased local pH,^50,51^ which destabilizes adsorbed intermediates and shifts the reaction environment in favor of undesired side reactions. Additionally, excessive cathodic polarization may induce changes in the catalyst’s morphology or electronic structure, further reducing CO_2_RR selectivity to ethylene.

The observed dependence of optimal operating potential on both the catalyst type and cell configuration emphasizes the complex interplay between reaction environment and material properties. While the characteristics and properties of CuO@C and CuONS are very similar and do not justify a change in mass transport properties, they exhibit a significantly different response to gas and liquid electrocatalytic tests, as reflected in the different impact on C_2_H_4_ FE. This result indicates that they exhibit different accessibility of CO_2_ and protons to the surface, which cannot be justified by mass transport limitations alone. There are effects related to surface hydrophobicity and charge accumulation that influence the response to the gas-electrolyte interface and local electric field effects.^52^ While it is not the objective to discuss these aspects in more depth here, as commented in the introduction, it is evident that this first example, together with the other examples presented later, demonstrates the key concept that surface intrinsic kinetics cannot be separated from the accessibility of reactants to the surface. Thus, it evidence that (i) the presence of specific active sites is not the only factor determining the pathways of transformation (the common assumption in the literature), and that consequently, (ii) in electrocatalysis, the intrinsic kinetics cannot be separated from mass transport of reactants (surface accessibility).

A more complex approach than that is usually utilized, which integrates considerations of interfacial electric fields or a spatially resolved characterization of the local electrochemical environment,^1^ would be necessary. However, current research and modeling of these reactions are far from providing quantitative, reliable approaches to describe these phenomena. Several studies have focused on the impact of cell configuration, for example, flow cell vs MEA systems, on variations in CO_2_ concentration and local pH gradients, as well as the selectivity of CO_2_RR to multicarbon products. None of them, however, is able to develop a generalized description. Furthermore, theoretical models and computational simulations have highlighted the influence of interfacial electric fields, which can modify the free energy of reactive intermediates and affect ion transport kinetics.^53–56^ However, these models and simulations are unable to describe the effects presented above and later accurately. They do not establish a proper link between FE, reaction pathways, and the surface accessibility of the reactants, which would allow for a quantitative description of the overall effects we report rather than specific effects. While a generalized approach and theoretical framework will certainly be useful, there are currently no developments in this area. This contribution would thus stimulate developments in this direction.

Overall, these findings reinforce the need to adopt a comprehensive approach, engineering not only the catalyst’s intrinsic properties but also tailoring the cell architecture to modulate the local reaction environment. In this context, the G-FC configuration emerges as particularly advantageous, not only promoting higher ethylene selectivity but also enabling operation under conditions that better mimic industrial electrolysis environments, characterized by low proton activity, high CO_2_ availability, and elevated current densities.

### 3.3 Influence of the Nature of the Electrolyte on Electrocatalytic Performance

The nature of the electrolyte has a significant influence on the reaction environment, interfacial chemistry, and overall catalytic performance.^57^ In particular, studying the effect of different electrolytes on the catalytic performance of electrocatalysts in the CO_2_RR to ethylene is critically important for several scientific and practical reasons due to the complexity of the required C–C coupling steps and the need for selectivity control.^58^

To investigate the effect of electrolyte composition on product selectivity and efficiency during CO_2_ electroreduction, four distinct testing conditions were evaluated using an L-FC configuration and two Cu-based electrocatalysts: CuONS and CuO@C. All experiments were conducted under identical conditions (CO_2_ flow of 12 mL/min and electrolyte flow of 15 mL/min) with an applied potential ranging from −1.4 to −2 V vs Ag/AgCl. In all considered conditions, K^+^ ions were selected due to their beneficial role in promoting C–C coupling during CO_2_ reduction. Larger cations like K^+^ can coordinate with *CO–CO intermediates, displacing water molecules and creating a localized hydrophobic environment. This reduces protonation of *CO, suppressing C_1_ product formation and favoring ethylene production. K^+^ also stabilizes key intermediates and enhances interfacial electric fields, making it well-suited for studying ethylene-selective CO_2_RR.^59^

Table 3 summarizes the electrolyte systems employed, which were deliberately selected to represent a range of mildly acidic to slightly basic environments while maintaining scalability and avoiding extreme pH values.

**Table 3:** Testing Conditions Studied in L-FC Cell Configuration for CuONS and CuO@C Electrocatalysts

condition	catholyte	anolyte	detected products
**1**	0.05 M H_2_SO_4_ + 3 M KCl	0.05 M H_2_SO_4_	H_2_, trace amounts of formic acid, ethanol, propanol
**2**	0.05 M H_2_SO_4_ + 1 M KCl	0.05 M H_2_SO_4_	H_2_
**3**	0.1 M KH_2_PO_4_/H_3_PO_4_ (pH = 2)	0.1 M H_3_PO_4_	H_2_
**4**	0.5 M KHCO_3_	0.5 M KHCO_3_	H_2_, C_2_H_4_

Conditions 1 and 2 employed diluted H_2_SO_4_ solutions containing KCl (3 and 1 M, respectively) as catholyte to enhance ionic conductivity. These conditions resulted predominantly in hydrogen evolution, with no ethylene detected in either condition. However, analysis of the liquid phase by ^1^H NMR (Figure S11) revealed the formation of C1 and C2 products such as formic acid, ethanol, and propanol, particularly under Condition 1. Moreover, the characterization of the electrocatalysts by XRD analysis confirmed the presence of KCl crystallization under Condition 1. This effect is less pronounced under Condition 2 due to the lower KCl concentration.

Condition 3 introduced a phosphate buffer (KH_2_PO_4_/H_3_PO_4_, pH = 2), simulating a mildly acidic environment. Similar to Conditions 1 and 2, ethylene was not detected, indicating that acidic conditions broadly disfavor the C–C coupling pathways required for ethylene generation on Cu-based catalysts. In contrast, Condition 4 utilized a slightly basic 0.5 M KHCO_3_ solution (pH ≈ 8.5). Under these conditions, ethylene formation was observed, albeit at a low FE of 4.7% at −2 V vs Ag/AgCl. The observed ethylene formation in slightly basic KHCO_3_ conditions (as seen in Condition 4) may, in part, be enhanced by the presence of K^+^ ions, which are known, as discussed above, to support C_2_ product generation. However, without sufficiently high pH or the formation of a copper hydroxide surface layer,^17,60,61^ the Faradaic efficiency for ethylene remains low, highlighting the multifactorial nature of CO_2_RR selectivity control. Moreover, it is essential to note that in all experiments, the electrolyte was continuously flowed through the cell to minimize local pH fluctuations at the electrode surface.^62–64^ As commented before, these results highlight the complexity of the interlinked phenomena and how the current design criteria and modeling approaches are unable to describe them properly.

### 3.4 Effect of Flow Field (FF) - Induced CO_2_ Distribution on Ethylene Selectivity

Efficient distribution of CO_2_ across the entire electrode surface is critical in CO_2_RR, particularly as electrode area increases. Uniform reactant distribution has a direct influence on product selectivity and system efficiency. This factor can also impact ethylene selectivity, as previous studies have shown that modifying the electrode to increase the contact time between intermediate CO and the catalyst enhances C2+ selectivity.^65^ There is an emerging interest in developing controlled flow fields at the electrode by applying a design to control the flow and distribution of CO_2_ at the electrode interface, particularly in zero-gap cell configurations.^66–70^

However, these studies do not clarify whether a better fluidodynamic design of the cell allows for reducing/avoiding mass transport limitations, or whether there are underlying more complex effects. In particular, when there is a separation between the mass transport effect and intrinsic kinetics, the reaction rate should be affected only by a decreased rate of access of CO_2_ to the surface, while C_2_H_4_ FE should remain unaffected.

To improve CO_2_ distribution to the electrocatalyst, we modified the cell configuration shown in Figure S8 by incorporating a flow field. A detailed description of the flow field used in our experiments, including its construction, geometry, materials, and integration into the electrochemical cell, is reported in the Supporting Information (Figure S12, which shows the details and characteristics of the FF used).

The flow field-integrated electrocatalytic cell configurations for both L-FC and G-FC systems are presented in Figure S13a and S13b, respectively, along with a detailed description of the flow field fabrication. Figure 4 presents the results for the two cell configurations (L-FC and G-FC) using the CuONS electrocatalyst, comparing performance with and without the flow field element. In all cases, the addition of the flow field increased the C_2_H_4_ FE despite the flow field design and CO_2_/electrolyte flow rates not being fully optimized.

**Figure 4:**
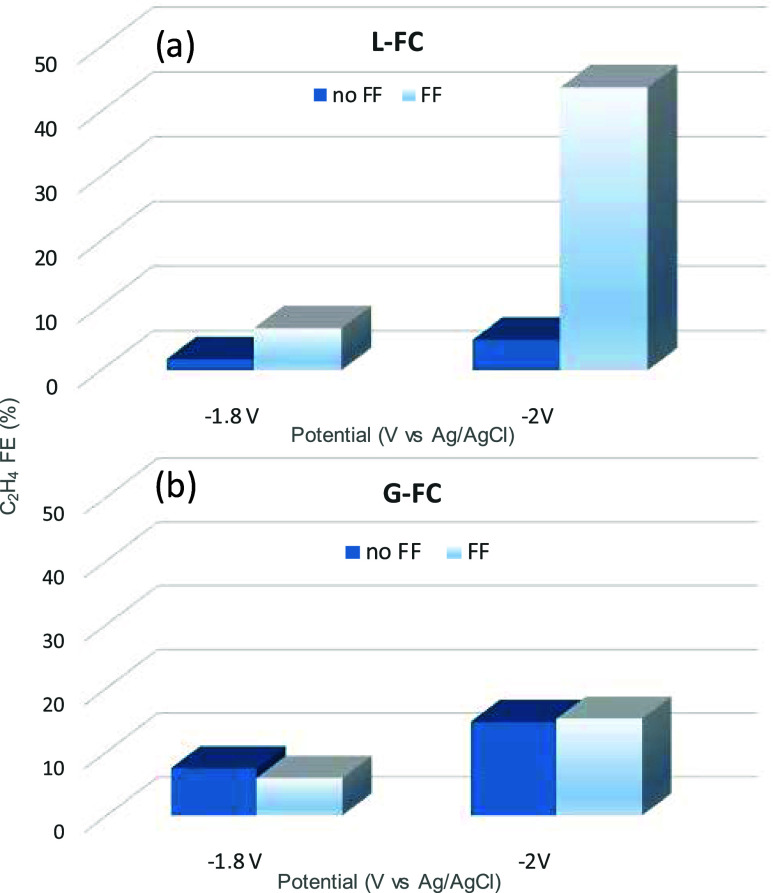
Comparison of the FE to ethylene for CuONS electrocatalyst in the L-FC (a) and G-FC (b) cell configurations with (FF) or without (no FF) the flow field element to force CO_2_ distribution. *Reaction conditions:* CO_2_ flow: 12 mL/min; Electrolyte flows: 15 mL/min, L-FC: 0.5 M KHCO_3,_ both anolyte and catholyte. G-FC 1 M KHCO_3_ anolyte.

The data presented in Figure 4 demonstrate that the use of a flow field significantly enhances C_2_H_4_ FE in the L-FC cell configuration. In fact, the incorporation of a flow field resulted in a substantial increase in C_2_H_4_ FE. Specifically, at −1.8 V vs Ag/AgCl, FE increased from 1.7% (no FF) to 6.5% (with FF), and at −2.0 V, from 4.7% (no FF) to 43.8% (with FF). In contrast, the G-FC configuration showed no significant enhancement in FE upon flow field integration.

However, reliance solely on FE values for performance evaluation can be misleading, particularly when operating current densities differ significantly. As FE represents the proportion of electrons contributing to a specific product, differences in total current can distort interpretations of catalytic activity and productivity. Specifically, the current density in L-FC increased from 3.1 to 38 mA/cm^2^ (at −1.8 V vs Ag/AgCl) and from 6.2 to 90 mA/cm^2^ (at −2.0 V vs Ag/AgCl). Similarly, in G-FC, current density rose from 5.8 to 27 mA/cm^2^ (at −1.8 V vs Ag/AgCl) and from 7.8 to 36 mA/cm^2^ (at −2.0 V vs Ag/AgCl). These variations prove that the improvement in FE is attributable to enhanced mass transport and system kinetics resulting from the flow field. Thus, a more robust comparison involves assessing the absolute ethylene production rate.

Figure 5 provides a comparison of ethylene production in mmol as a function of current density, offering a clearer representation of system productivity. The data reveal a direct correlation between current density and C_2_H_4_ output, with the flow field consistently improving ethylene production across both L-FC and G-FC configurations. The FF-integrated L-FC displayed the highest productivity, especially at elevated current densities, highlighting the critical role of enhancing CO_2_ distribution on the electrode surface. Compared to systems lacking a structured gas distribution layer, the presence of a well-designed FF offers several key advantages.

**Figure 5:**
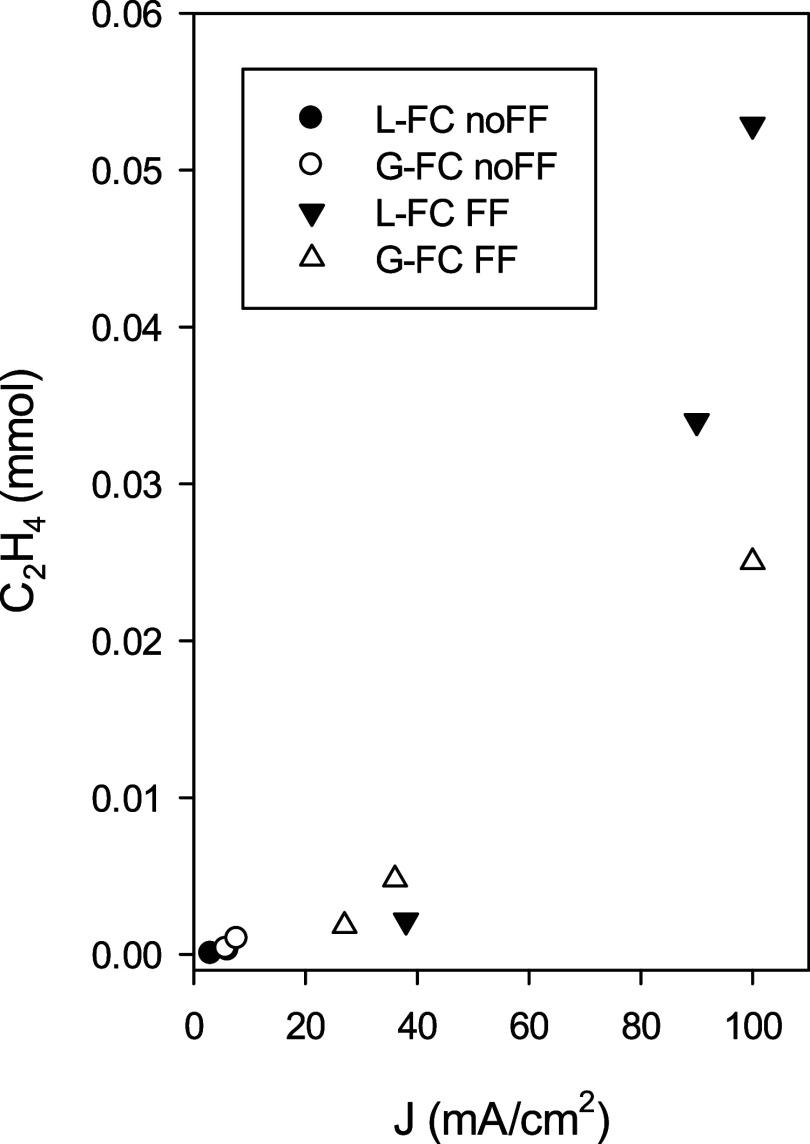
C_2_H_4_ production (mmol) as a function of current density (mA/cm^2^) under different cell configurations: L-FC noFF (●), G-FC noFF (○), L-FC FF (▼), and G-FC FF (Δ).

The FF promotes uniform CO_2_ distribution across the active area of the GDE.^66,67,71^ As shown in various studies, this uniformity is essential in CO_2_RR, where continuous and even well-distribution of CO_2_ to the catalyst layer is required to sustain high reaction rates and product selectivity. In the absence of an FF, CO_2_ may stagnate or follow preferential pathways, resulting in mass transport limitations, inhomogeneous reaction zones, and decreased overall performances.^10,72^ The FF facilitates the efficient removal of both gaseous (e.g., H_2_ or C_2_H_4_) and liquid (e.g., condensed ethanol or flooded electrolyte) species, which could otherwise accumulate and obstruct catalytic sites. These explanations, however, do not properly describe our experimental results and the market influence of C_2_H_4_ FE as well as dependence on cell configuration.

### 3.5 Role of the Ion Exchange Membranes

Ion exchange membranes (IEMs) play a critical role in the CO_2_RR, as they prevent electronic short-circuiting between electrodes, limit cross-contamination by physically separating the cathodic and anodic compartments, and ensure ionic continuity by selectively transporting charge carriers. Beyond these classical roles, the membrane can also influence the local electrochemical environment, particularly when the catalyst is in direct contact with it, as in MEA configurations. To investigate the role of the IEM’s nature on the electrocatalytic performance of the electrocatalyst, we compared the commonly used cation exchange membrane Nafion 115 ^73–75^ with the anion exchange membrane FUMASEP-FAA-3PK-75.^76–79^

We thus selected for this study two commonly used types of membranes from several literature studies, which have opposite characteristics. Nafion 115 is a widely used protonic membrane. The Fumasep FAA-3-PK-75 is a polyketone (PK)-reinforced anion exchange membrane characterized by low resistance, high selectivity, high mechanical stability, and excellent stability in both alkaline and acidic environments. The thickness is 75 μm, similar to that of the Nafion membrane. The transport mechanisms in these two membranes are different, and thus their testing provides further indications for the objective of this study.

The implementation of the Fumasep FAA-3PK-75 anion exchange membrane in both L-FC and G-FC configurations led to a substantial enhancement in electrochemical performance. Specifically, the current density increased by a factor of 3 in the L-FC configuration, while it nearly quadrupled, reaching values close to 30 mA/cm^2^ in the G-FC configuration. Correspondingly, C_2_H_4_ FE rose from 4.7 to 9.7% in L-FC. Although ethylene production also increased in the G-FC configuration, the elevated current densities led to a moderate decline in C_2_H_4_ FE, decreasing from 14.7 to 12% (Figure 6).

**Figure 6:**
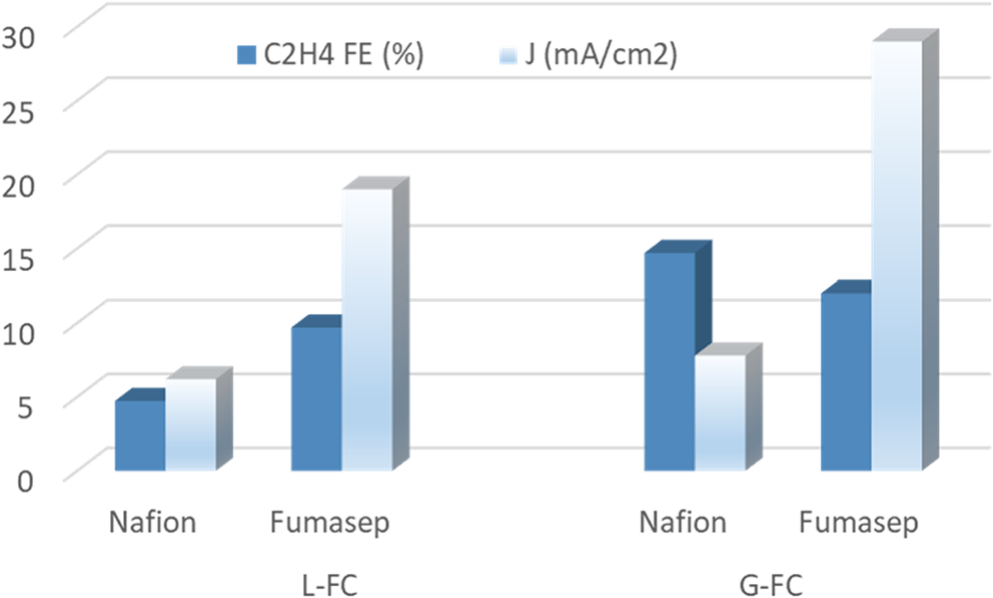
Comparison of C_2_H_4_ FE, % and current density (J, mA/cm^2^) for L-FC and G-FC configurations using Nafion and Fumasep membranes. *Reaction conditions:* CO_2_ flow: 12 mL/min. Electrolyte flow: 15 mL/min, L-FC: 0.5 M KHCO_3_ for both anolyte and catholyte (Nafion), 0.5 M KHCO_3_ for catholyte 1 M KOH for anolyte (Fumasep). G-FC 1 M KHCO_3_ (Nafion) e 1 M KOH (Fumasep)

These enhancements can be primarily attributed to the local chemical environment at the electrocatalyst-membrane interface. The Nafion membrane allows proton migration from the anolyte, leading to a decrease in the pH of the catholyte and favoring the competitive HER. The use of anion exchange membranes results in a more alkaline microenvironment, providing lower polarization losses and thus higher limiting current densities compared to cationic membranes.^31,76^ However, this interpretation is insufficient to describe our results, as well as the significant dependence of C_2_H_4_ FE on the type of membrane and cell configurations, and the impact of current density on C_2_H_4_ FE. Current design criteria for electrocatalysts and theoretical approaches, as well as considerations on mass transfer limitations, are unable to properly describe the results and the effect, for example, of current density.

By increasing the applied potential to −2.0 V, corresponding to a current density of 63 mA/cm^2^, the C_2_H_4_ FE reaches approximately 25 and 30% when the current density reaches 100 mA/cm^2^. These FE values discussed here were calculated using eq 3, based on the measured ethylene production (in mmol) and the corresponding current density values reported in Figure 7.

**Figure 7:**
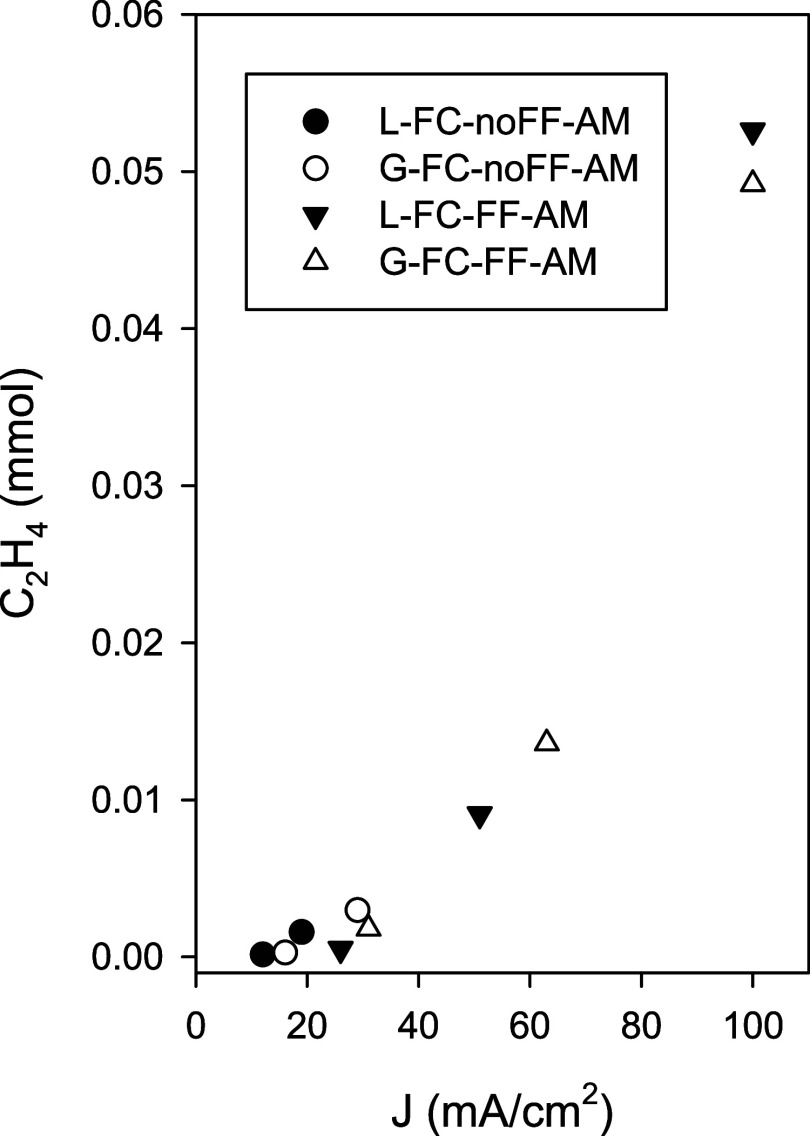
C_2_H_4_ production (mmol) as a function of current density (mA/cm^2^) under different cell configurations by using Fumasep anionic membrane: L-FC noFF (●), G-FC noFF (○), L-FC FF (▼), and G-FC FF (Δ).

We further analyzed the relationship between ethylene production and current density using the anion exchange membrane in both L-FC and G-FC configurations, with and without the application of a flow field (Figure 7). The data clearly demonstrate that ethylene production increases with current density across all configurations, with a notable increase only observed when the current density exceeds 50 mA/cm^2^. The integration of FF in both L-FC and G-FC cells, in the presence of the anion exchange membrane, significantly enhances the current density, achieving the highest ethylene production at approximately 100 mA/cm^2^. This synergy enhances hydroxide ion transport, contributing to a more favorable reaction environment and increased CO_2_ availability, which enables the system to reach much higher current densities. This effect is most pronounced in the G-FC cell configuration.

As mentioned above, current interpretations are unable to explain these effects overall, and evidence clearly indicates that separating mass transport from intrinsic kinetics is incorrect in electrocatalysis. The results could be interpreted in terms of factors determining the surface accessibility of the reactants (CO_2_, protons) to the catalyst, but these factors are not only determined by classical aspects of mass transport, nor by considerations of the local microenvironment, pH, and similar effects. They cannot explain the clear effect of current density commented on above.

It is worth noting that the introduction of the FF had a more pronounced effect on ethylene production in the L-FC configuration when using the Nafion cation exchange membrane (Figure 5). The effect is thus not dependent on the nature of the membrane or its potential modification of the electrocatalyst’s nature. Together with the previous result, it further demonstrates the determining role of cell configuration in the electrocatalyst and, thus, the role of the microenvironment at the electrocatalyst surface.^22,23,80^ The electrocatalyst characteristics, including modifications to change accessibility, wettability (hydrophobicity), CO_2_-philicity, nanoconfinement and other properties,^13,33,80–86^ combine with the cell/electrode design (and related influence on transport properties) in determining the effective microenvironment and thus the FE to ethylene.

### 3.6 The Role of Current Density

The majority of tests in CO_2_ electroreduction are performed by varying the applied potential and measuring the current density obtained, which strongly depends on the resistances present and, thus, the specific cell and electrode characteristics. However, what happens when the tests are performed in a given cell/electrode assembly by fixing the current density instead? By forcing the CO_2_RR reaction rate, surface accessibility becomes even more controlling, while the higher charge of the electrocatalyst also affects the transport properties.

We emphasize surface accessibility, rather than mass transport limitations, because it is a distinct concept from classical transport limitations related to fluid dynamic considerations, even when an electrical field is present. In addition, since these aspects determining C_2_H_4_ FE are present in both liquid and gas-phase cell configurations, it is clearly not related to gas solubility or the classical double layer.

The experimental results reveal an unexpected effect: tests at high current densities can impact selectivity. However, this is an effect that has been scarcely investigated and may eventually be expected to be negative, i.e., selectivity decreases at higher current densities. This type of test could provide further indications of the key parameters determining the FE to ethylene. For this purpose, CO_2_RR tests were performed under galvanostatic conditions at a constant current density of 100 mA/cm^2^. The electronic and ionic conductivities were modulated by adjusting the electrolyte composition in both the catholyte and anolyte compartments for the L-FC configuration, and in the anolyte compartment for the G-FC configuration, as well as by varying the membrane type.

Table 4 presents a comparative analysis of C_2_H_4_ FE using Nafion and Fumasep membranes under both galvanostatic conditions (100 mA/cm^2^) and at a fixed potential of −2 V vs Ag/AgCl, in both L-FC and G-FC configurations. The data show that operating under a constant current density yields significantly higher FE values than those achieved at a fixed potential, indicating that galvanostatic control allows for more efficient electron utilization across both membrane types.

**Table 4:** Comparison of C_2_H_4_ FE between L-FC and G-FC Configurations Using Nafion and Fumasep Membranes at Different Operating Conditions

		C_2_H_4_ **FE (%)**	
		**L-FC**	**G-FC**
**Nafion**	100 mA/cm^2^	61.3^a^	28.9^b^
	–2 V (vs Ag/AgCl)	43.7^a^	15.2^b^
		(90 mA/cm^2^)	(36 mA/cm^2^)
**Fumasep**	100 mA/cm^2^	60.9^c^	56.9^e^
		67.0^d^	
	–2 V (vs Ag/AgCl)	20.7^c^	25^e^
		(51 mA/cm^2^)	(63 mA/cm^2^)

a 0.5 M KHCO_3_/0.5 M KHCO_3_.

b 1 M KHCO_3_.

c 0.5 M KHCO_3_/1 M KOH.

d 1 M KOH/1 M KOH.

e 1 M KOH.

In the L-FC, Nafion achieves a C_2_H_4_ FE of 61.3% under galvanostatic conditions, which is markedly higher than the 43.7% recorded at −2 V. In the G-FC, Nafion reaches an FE of 28.9%, nearly double the 15.2% observed at −2 V, where the associated current density drops to 36 mA/cm^2^. However, in the tested conditions (where anolyte concentration is higher than that reported before, i.e., 1 M instead of 0.1 M of KHCO_3_, and the reaction rate is forced by operating at a fixed current density of 100 mA/cm^2^), there is a deposit of carbonate salts on the electrode (Figure S14 reporting the postuse characterization of CuONS-based GDE in G-FC setup using Nafion membrane).

Fumasep, by contrast, demonstrates strong performance in both configurations under galvanostatic operation, achieving 60.9% in L-FC and 56.9% in G-FC. These results underscore the superior overall performance of the Fumasep membrane, particularly in the G-FC configuration, when compared to Nafion. Unlike what was observed with the use of the Nafion membrane in G-FC, no carbonate deposits were found when the anionic membrane was used. While Nafion performs best in L-FC, the results also highlight that the anion exchange Fumasep membrane offers promising efficiency even in L-FC configurations.

A critical factor influencing this behavior is the composition of the Electrolyte. Nafion shows optimal performance in KHCO_3_-based systems, consistent with its proton-conducting properties, whereas Fumasep excels in alkaline environments such as 1 M KOH, in line with its hydroxide transport mechanism. The use of KOH significantly enhances C_2_H_4_ selectivity, likely due to its ability to suppress the hydrogen evolution reaction (HER) and increase the availability of CO_2_ at the catalyst surface.

The results of Table 4 indicate that, in the tested conditions, (i) the Nafion membrane works better in the L-FC cell configuration; (ii) the Fumasep membrane works well both in L-FC and G-FC cell configurations; and (iii) overall, the L-FC system works better than G-FC. The latter indication is thus the opposite of what was previously observed under different operating conditions. Further relevant indications are that

- High current densities favor the production of ethylene in both G-FC and L-FC configurations, with FE to ethylene >60% at high current density and thus in line with those reported in the literature (Figure 2), even if the interpretation is different (role of the cell/reaction conditions rather than of the electrocatalyst).
- The best performances in terms of C_2_H_4_ FE at fixed current density (100 mA/cm^2^) were achieved in the L-FC configuration, while the opposite was observed in the previous tests.
- The protonic membrane (Nafion) yields a better FE in the L-FC configuration with a 0.5 M KHCO_3_ catholyte, while the anionic membrane (Fumasep) performs similarly in both configurations.
- C_2_H_4_ FE improves when using 1 M KOH as the catholyte in L-FC configurations with the anionic membrane; these conditions are milder than those typically reported in the literature for achieving the best FE results.

After comparing all the data, optimizing the electrolyte, and applying current/potential, the best performances (about 67% FE toward ethylene) were obtained for the CuONS electrocatalyst in the L-FC cell configuration using the anionic membrane with a KOH 1 M anolyte and applying a fixed current density of 100 mA/cm^2^.

## 4 Conclusions

The comparison of a set of copper-based electrocatalysts synthesized according to those previously reported in the literature as highly active for CO_2_RR to ethylene reveals that when tested under the same experimental conditions, their performances differ significantly from those reported in the literature under optimized conditions. This fact raises the crucial question of whether the behavior, particularly the selectivity for ethylene and thus the dominant surface pathways of transformation, is determined by the nature of the active sites or is largely influenced by other factors. The related consequences regard the reliability of mechanistic and design aspects in maximizing selectivity.

To address this question, we have thus tested a single catalyst among those selected from the literature (a copper oxide nanosheet catalyst - CuONS) under an extended range of extrinsic factors, such as cell configuration, type of membrane, and field flow. Additional tests were also conducted using a second catalyst (CuO@C). We use the term extrinsic to differentiate the parameters investigated that differ from the intrinsic catalyst factors, such as the type of active sites present, which are usually considered in mechanistic and design considerations. However, catalysts may differ not only in terms of the nature of the active sites, but also in other properties, such as the surface electrical field under the application of a potential, hydrophilicity, etc., which may also determine the accessibility of the reactants to the catalyst surface, and in turn selectivity and surface pathways of transformation. There is an interlink between extrinsic factors and these other catalyst characteristics, which determines the crucial aspect, typically not considered, that the intrinsic kinetic cannot be separated from mass transport limitations in terms of classical dependence on fluidodynamic local situations. We emphasize in the discussion the concept of surface accessibility, rather than mass transport limitations, because it is a distinct concept from classical transport limitations related to fluid dynamic considerations, even when an electrical field is present.

The obtained results evidenced that C_2_H_4_ FE can range from 0 to nearly 70%, thus in a range largely beyond that which may be expected from the presence of only diffusional limitations overlapping with the intrinsic kinetic behavior. This concept is the typical assumption in the literature that cell and electrode design can mainly influence the rate of CO_2_ diffusion to the catalyst.^87,88^ In this sense, it is an extrinsic factor distinct from the intrinsic nature of the catalyst. While there are attempts to discuss the complex electrochemical interfaces,^89^ and how microenvironment engineering influences selectivity,^83^ it is still surprising that the underlying concept of separation between intrinsic factors determining the kinetics and extrinsic factors that determine the local concentration of reactants has never been verified and validated.

Thus, the catalyst is designed and optimized in terms of the nature of the active sites, which determine the aimed surface pathways of the reaction. Meanwhile, the cell and electrode design are separate aspects that can be considered independently, as they involve determining only the mass transfer and a few other related aspects.

Develop a new model and approach going beyond this assumption and accounting for the very complex nature of reactive electrocatalytic surface is largely beyond the scope of this manuscript, which limit to evidence that the intrinsic nature of the electrocatalysts, i.e., the presence of specific sites and reaction mechanisms, is largely overlooked by extrinsic factors that determine the effective dynamic concentration of CO_2_, protons, and intermediates at the surface. The main result of this study, i.e., that the C_2_H_4_ FE may change from zero to 70% or more by changing the testing conditions, proves this crucial question. It has also been proven that intrinsic and extrinsic factors are highly interlinked; thus, they cannot be optimized separately.

For this reason, the strong effect of parameters such as operating at a fixed current density or using field-flow configurations that are not commonly investigated but have a major impact on performance. Cell configuration, i.e., operations in G-FC or L-FC modes, also has an important impact; however, the preferred cell configurations strongly depend on the other parameters. Thus, a general indication cannot be given; however, the results highlight how this aspect, often overlooked, is also an important element in determining the C_2_H_4_ FE.

An important aspect that is evident is that operations at high current densities significantly improve the C_2_H_4_ FE. Thus, tests performed under cell configuration types such as H-cell, which do not allow for reaching high current densities due to their intrinsic design, are not representative of the effective behavior and may give misleading results even in a screening phase. Combining high FE and current density is the industrial objective, but this depends only secondarily on the type of electrocatalyst. At the same time, the dominant effect is the effective population of CO_2_ and protons/electrons near the electrocatalyst surface. This observation also raises a question about many mechanistic studies which do not account for this critical aspect.

The interlinked dependence of intrinsic and extrinsic parameters is due to the double role of the electrocatalyst, which is certainly important in terms of specific sites. Still, it plays a role (often neglected and not accounted for in mechanistic assessments) in determining the effective microenvironment and concentration of species at the electrocatalytically active surface. In fact, despite similar ECSA values, significant differences in performance persisted, confirming that catalyst surface area alone does not explain the observed outcomes. These results highlight the significant impact of various operational parameters on electrocatalytic behavior. It also provides evidence that metrics such as ESCA are unsatisfactory because they are based on an implicit separation between intrinsic and extrinsic factors.

These aspects are often determined under conditions far from those effectively in operation during CO_2_RR, highlighting the need for effective methodologies that provide the right mechanistic information. Although this study aimed to demonstrate the crucial role of various electrode/cell design aspects and how they synergistically interact with reaction conditions in determining performance, aspects often not well identified in the literature, it also sheds light on important mechanistic considerations for improving the C_2_H_4_ FE in CO_2_RR.

## Supplementary Material



## Notes

Results on the synthesis and characterization of the electrocatalysts, the experimental procedure, the membranes used, the cell setup with details including the fabrication of the flow field, and additional electrochemical characterization data (PDF)
