# Supplementary material for: CO2 Electroreduction to Ethylene: The Determining Role of the Cell and Electrode Design
Source: Energy Fuels. 2025 Sep 5;39(37):17899–912. doi: 10.1021/acs.energyfuels.5c02802 (PMC12451666; doi:10.1021/acs.energyfuels.5c02802)
Supplement: Supplementary file 1 [file ef5c02802_si_001.pdf]

# $CO_2$ electroreduction to ethylene: the determining role of the cell and electrode design

Paolo Squillaci[a], Georgia Papanikolaou*[a], Siglinda Perathoner[a], Gabriele Centi[a], Mattia Melloni[b], Angelo Ferrando[b] and Paola Lanzafame*[a]

[a] *Department ChiBioFarAm - Industrial Chemistry, University of Messina, INSTM CASPE (Laboratory of Catalysis for Sustainable Production and Energy) and ERIC aisbl, Viale F. Stagno d'Alcontres 31, Messina, 98166, Italy.*

[b] *Versalis SpA, Basic Chemical & Plastics Research Centre, Via Taliercio 14, 46100 Mantova, Italy.*

# SUPPLEMENTARY INFO

**Synthesis of the electrocatalysts**

**CuO@C**: The catalyst was synthesized via the sol-gel method [1]. First, 3 g of polyvinylpyrrolidone (PVP) was completely dissolved in 90 mL of deionised water in a round-bottom flask under vigorous stirring. Subsequently, 4.5 g of copper nitrate was added to the solution and stirred until it was fully dissolved. The resulting solution was then heated at 95%˚C for 1 hour, during which a colour change from light blue to green was observed, indicating the formation of a complex between copper and PVP. The mixture was then dried, transitioning through a gel-like state until a spongy solid was obtained. This solid was finely ground using a mortar and pestle to yield a fine green powder.

**CuAl**: The catalyst was synthesized via a wet chemical reduction method with minor modification [2]. Briefly, 4 mL of 0.9 M sodium citrate tribasic was added to 300 mL of distilled water while stirring. Then, 2%mL of 1.2%M copper sulfate and 2%mL of 0.12%M aluminium nitrate were added to the solution and stirred for 10 minutes to achieve a Cu: Al molar ratio of 10:1. Subsequently, the metal salts were converted into their corresponding hydroxides by rapidly injecting 2%mL of 4.8%M sodium hydroxide under continuous stirring. The hydroxides were then reduced to their respective oxides by adding 2.0 mL of a 1.2 M L-(+)-ascorbic acid solution under stirring, which caused the precipitation of a solid. Finally, the precipitate was filtered, washed with both water and ethanol and dried under vacuum at 60%˚C.

**$Cu/Cu_2O$**: The catalyst was synthesised via an aqueous precipitation method [3]. Briefly, 1.25 g of copper sulfate and 6 g of polyethene glycol were sequentially dissolved in 50 mL of distilled

water under stirring, resulting in a gradual colour change from transparent to light blue and finally to a white-opalescent colour. Subsequently, 100 mL of an alkaline solution containing 0.9 g of L-(+)-ascorbic acid and 0.4 g of sodium hydroxide was added to the mixture under continuous stirring for 30 minutes, during which the colloidal solution turned orange. Then, 50 mL of distilled water containing 0.4 g of sodium borohydride was added to the orange solution, inducing the reduction of copper species and changing the solution's colour to dark red. The suspension was aged overnight, and the resulting catalyst was filtered, washed, and dried under a vacuum at 60%˚C.

**CuNWs**: The catalyst was prepared via a wet assembly chemical reduction method [4]. First, 184%mg of copper nitrate was completely dissolved in a sodium hydroxide solution (15%M) under vigorous stirring. Subsequently, 1.2 mL of ethylenediamine (2.5 mmol) was added to the mixture under continuous stirring. The resulting solution was then heated to 70%˚C for 3 minutes. Afterwards, 66 μL of hydrazine (64% in water) was added, and after complete homogenization, stirring was stopped to facilitate the formation of the nanowires. During this process, the blue solution transitioned through a colourless phase before turning dark red. The floating nanowires were separated from the liquid phase using a separation funnel by extraction with 250 mL of an aqueous solution containing 0.0075 g of polyvinylpyrrolidone and 20 μL of diethylhydroxylamine. The collected solid was washed with the same solution, and finally, it was dried under a vacuum and stored.

**CuMOF**: The catalyst was prepared via a pyrolytic method [5]. Briefly, 2%g of commercial Basolite C300 was placed in a quartz tube reactor. After degassing for 1 hour under a nitrogen flow, the sample was pyrolysed at 400%˚C for 4 hours with a heating rate of 2%˚C/min, maintained under a constant nitrogen flow of 40%mL/min to ensure anoxic conditions. Finally, the sample was collected and stored under a vacuum.

**Ni-CuNWs**: The Ni catalyst was prepared via a hard-template polymerisation method [6]. Briefly, SBA-15 was synthesized as previously reported [7]. A solution containing 288 mg of nickel acetylacetonate, 8 mL of ethylenediamine, and 10 mL of carbon tetrachloride was prepared and stirred for 30 minutes to ensure complete homogeneity. Subsequently, 3.2 g of SBA-15 was added to the above solution under continuous stirring, and the mixture was heated at 90˚C for 16 hours under reflux conditions. The temperature was then increased to 120%˚C for 4 hours to obtain a solid. Finally, the resulting powder was pyrolysed in a quartz tube at 800%˚C for 2 hours (heating rate: 1%˚C/min) under anoxic conditions, with a constant argon flow of 50 mL/min. The Ni-CuNWs catalyst was obtained by mixing the correct amount of CuNWs and Ni catalysts using a mortar and pestle to achieve a Cu:Ni ratio of 10:1.

**Morphological and structural characterization**

The catalyst's morphologies and atomic composition were examined by scanning electron microscope (SEM) and energy-dispersive X-ray (EDX) analysis using a PhenomProX Scanning Electron Microscope with accelerating voltages of 10 kV and 15 kV, respectively. Powder X-ray diffraction (XRD) analysis was conducted by a Bruker D2 Phaser diffractometer equipped with

Cu K α radiation (λ= 1.54056 Å).

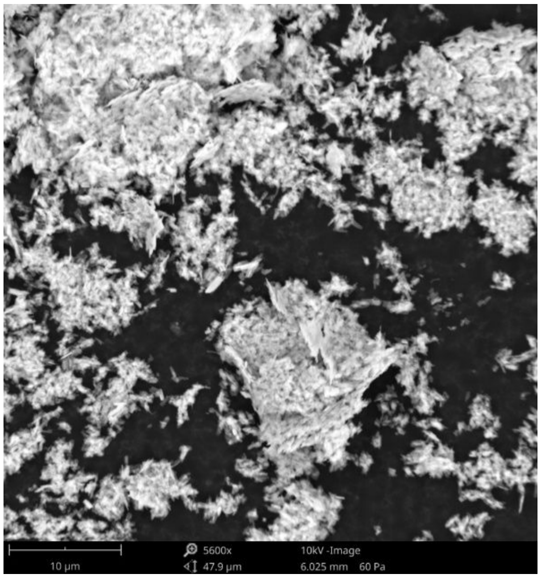


| EDX | Cu | O |
|---|---|---|
| Atomic Concentration (%) | 48 | 52 |

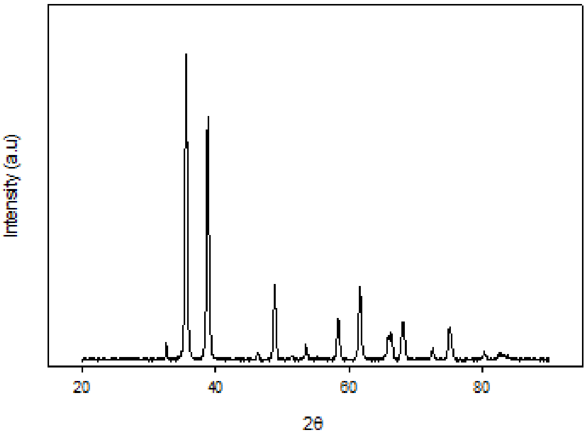


**Figure S1:** Morphological, chemical and structural characterization of CuONS

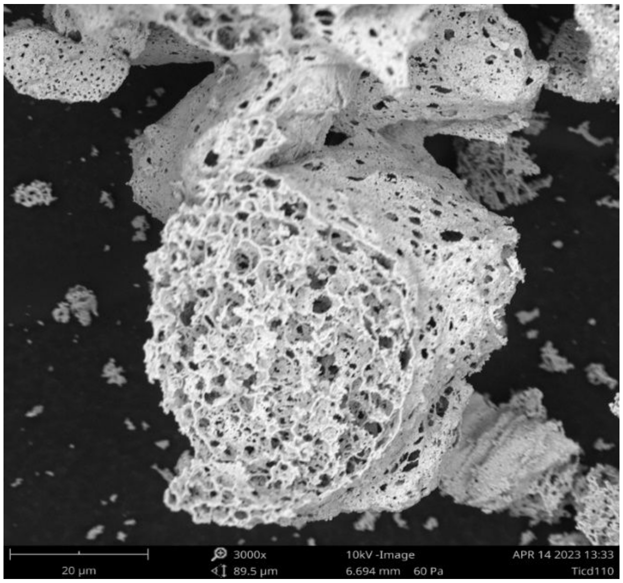


| EDX | Cu | O | C |
|---|---|---|---|
| Atomic Concentration (%) | 53.6 | 43 | 3.4 |

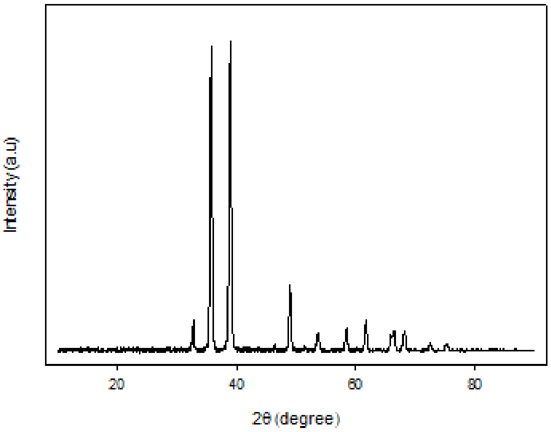


**Figure S2:** Morphological, chemical and structural characterization of CuO@C

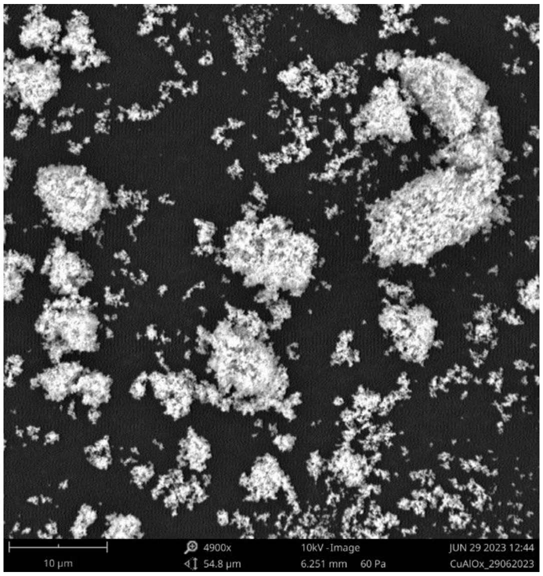


| EDX | Cu | O |
|---|---|---|
| Atomic Concentration (%) | 58.1 | 41.9 |

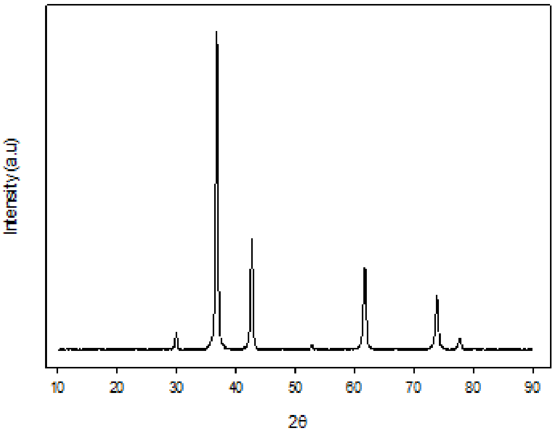


**Figure S3:** Morphological, chemical and structural characterization of CuAl

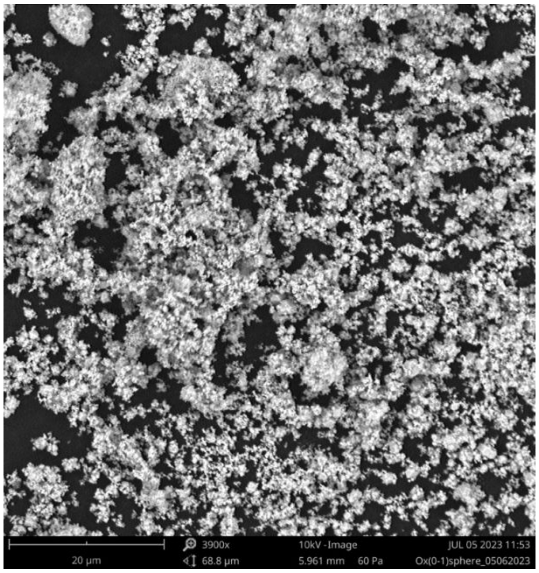


| EDX | Cu | O |
|---|---|---|
| Atomic Concentration (%) | 64.1 | 35.9 |

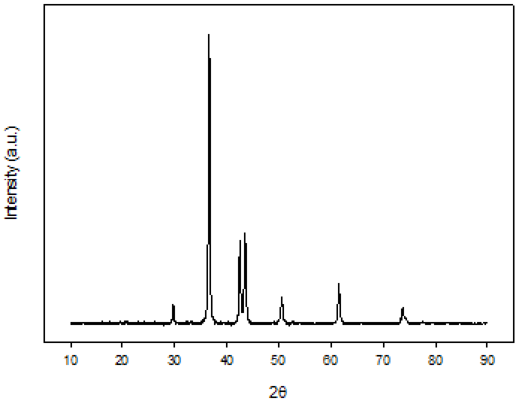


**Figure S4:** Morphological, chemical and structural characterization of $Cu/Cu_2O$

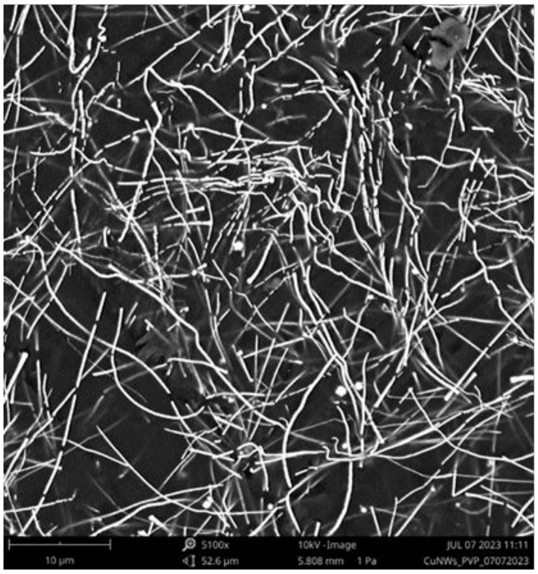

| EDX | Cu | O |
|---|---|---|
| Atomic Concentration (%) | 52.2 | 47.8 |

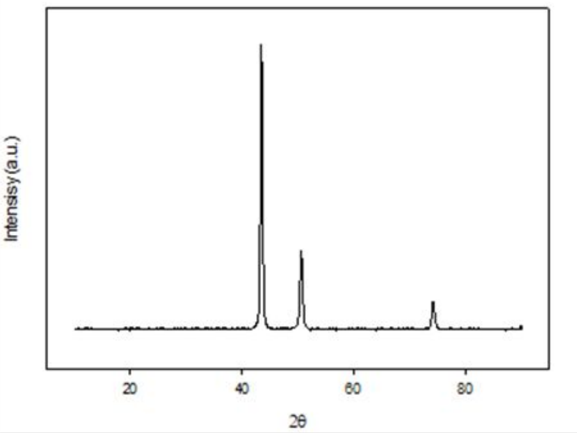


**Figure S5:** Morphological, chemical and structural characterization of CuNWs

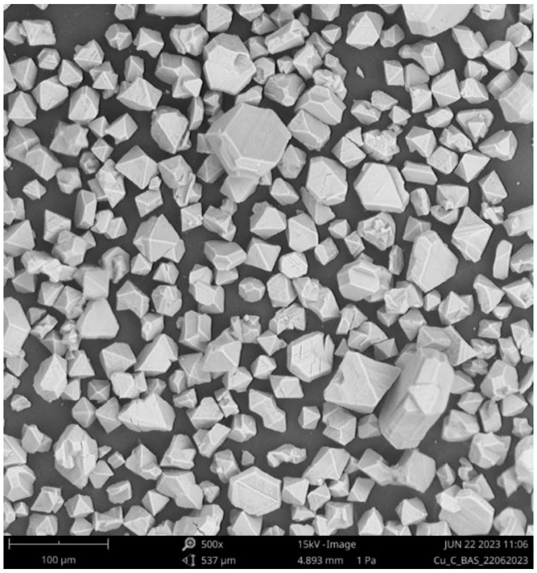

| EDX | Cu | O | C |
|---|---|---|---|
| Atomic Concentration (%) | 81.1 | 11.9 | 7 |

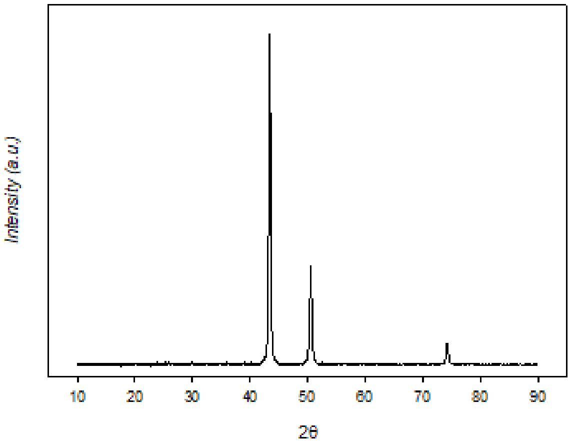

**Figure S6:** Morphological, chemical and structural characterization of CuMOF

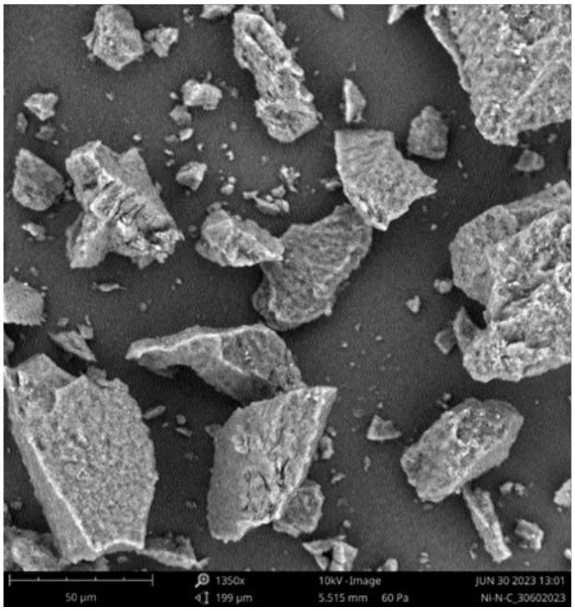


| EDX | Cu | Ni | O | C |
|---|---|---|---|---|
| Atomic Concentration (%) | 32.2 | 3.1 | 41.8 | 22.9 |

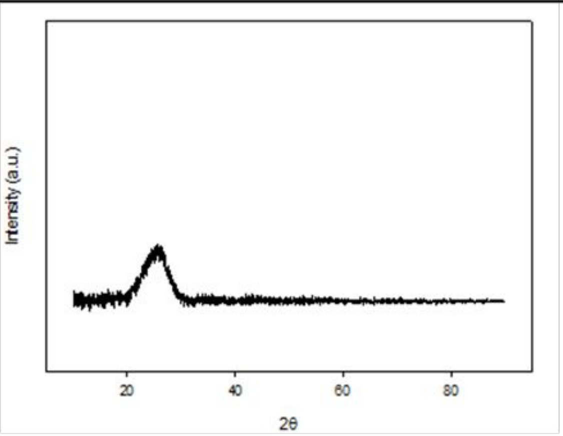


**Figure S7:** Morphological, chemical and structural characterization of Ni-CuNWs

**Activation of the cation and anion exchange membrane**

The Nafion 115 membrane was initially purified by treatment with $H_2O_2$ at 80°C for 1 hour. Subsequently, the membrane was converted to the $H^+$ form by treatment with 0.5 M sulfuric acid under the same conditions. The activated membranes were then rinsed with ultrapure water to remove any excess acid and stored in a closed container.

The Fumasep FAA-3PK-75 membrane was initially completely immersed in NaCl for 24 hours to remove any impurities. Subsequently, the membrane was activated in the $OH^-$ form by immersing it in a 1 M KOH solution at room temperature for 2 days. The activated membrane was then stored in a tank filled with 1 M KOH to prevent contamination from atmospheric $CO_2$.

**Setup of electrocatalytic cell**

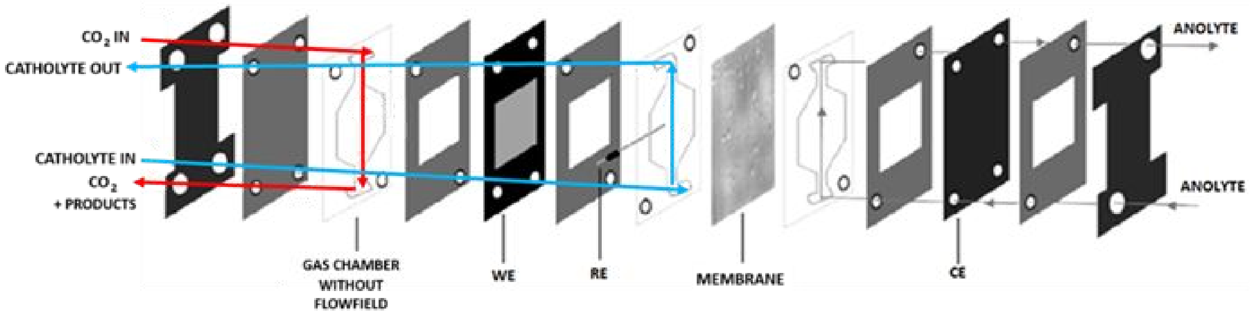


**Figure S8:** Schematic diagrams of the micro flow electrocatalytic cell

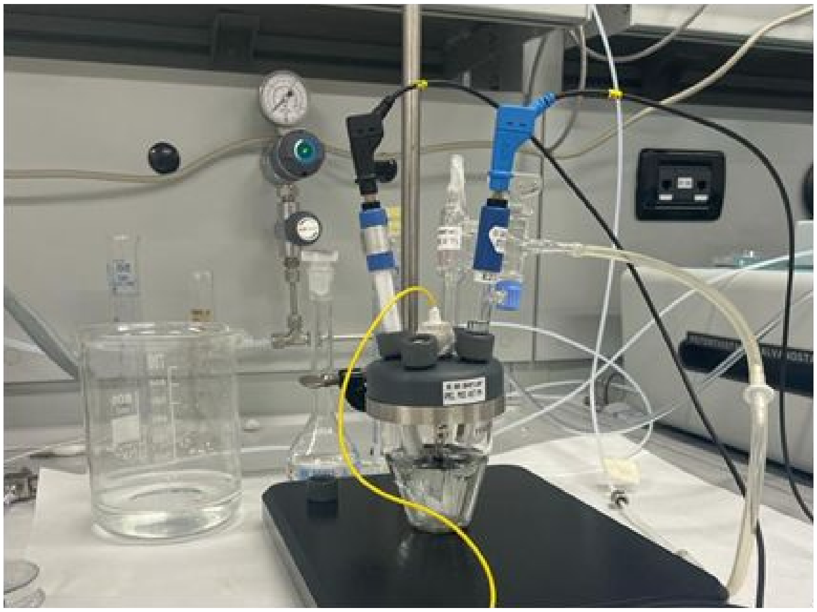

**Figure S9:** Three-electrode system for the electrochemical characterization

**Table S1** Comparison of the performances of the synthesized electrocatalysts in the G-FC cell setup (*Reaction conditions*: $CO_2$ flow: 12 mL/min; electrolytes flow: 15 mL/min, 1M $KHCO_3$ (anolyte), Nafion115 membrane)

| *Electrocatalyst* | *FE% to ethylene* | | | *FE% to CO* | | | *J(A/cm²)* | | |
|---|---|---|---|---|---|---|---|---|---|
| | *-1.6V* | *-1.8V* | *-2.0V* | *-1.6V* | *-1.8V* | *-2.0V* | *-1.6V* | *-1.8V* | *-2.0V* |
| CuONS | - | 13.6 | 9.9 | 26.8 | 0.1 | 0.1 | 0.005 | 0.010 | 0.011 |
| CuO@C | 3.6 | 10.7 | 0.01 | 27.1 | 6.8 | 0.01 | 0.003 | 0.011 | 0.011 |
| CuAl | 0.3 | 1.6 | 7.2 | - | 17.1 | 17.1 | 0.028 | 0.017 | 0.015 |
| Cu/$Cu_2O$ | - | 0.5 | 8.5 | 6.6 | 8.5 | 11.7 | 0.008 | 0.017 | 0.017 |
| CuNWs | - | 0.8 | - | 0.01 | 5.5 | 0.1 | 0.002 | 0.027 | 0.023 |
| CuMOF | - | 3.5 | 1.8 | 0.1 | 12.5 | - | 0.003 | 0.013 | 0.005 |
| Ni-CuNWs | - | - | - | 87.1 | 93.2 | 0.02 | 0.005 | 0.006 | 0.008 |

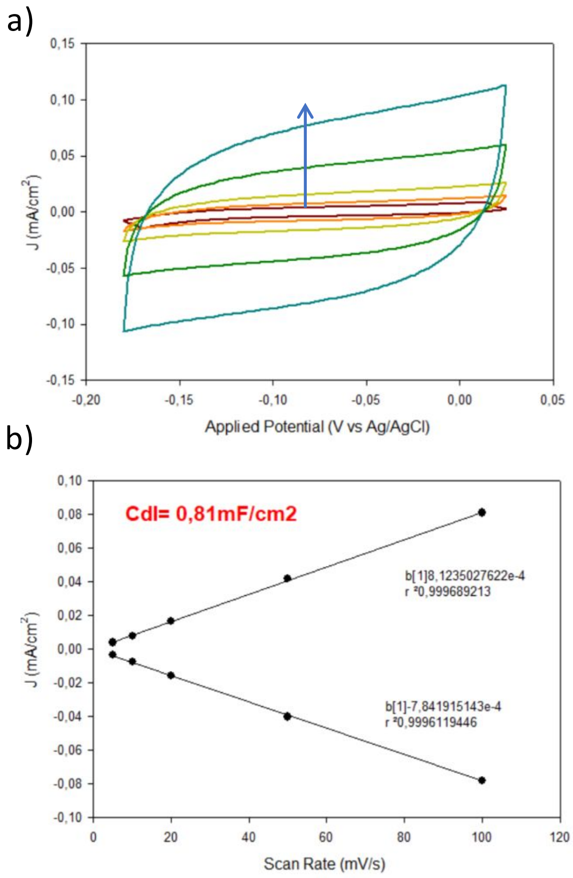


**Figure S10:** a) Cyclic Voltammetry curves recorded at different scan rates in the non-Faradaic

region; b) Electrochemical double layer capacitance ($C_{dl}$)

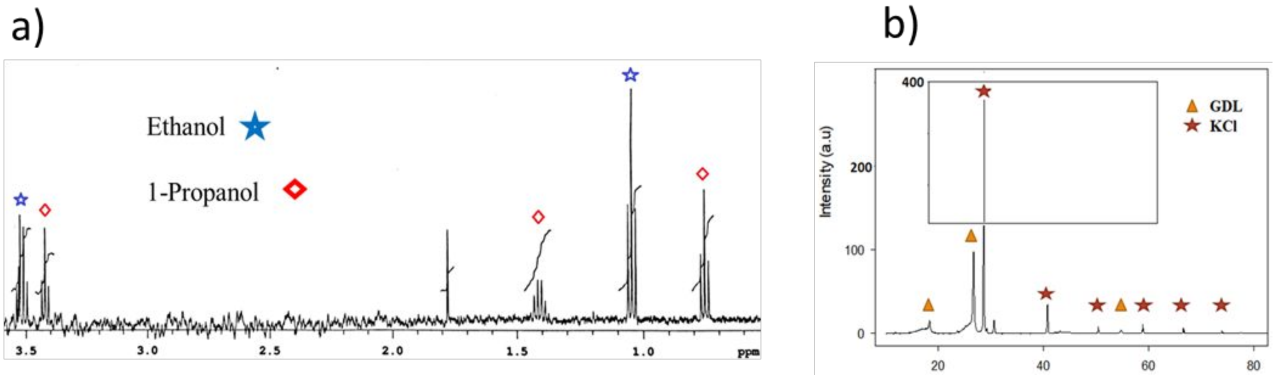


**Figure S11:** $H^1$-NMR of liquid products (a) and XRD characterization of GDL after electrocatalytic test

**Fabrication of flow field (FF)**

The flow field (FF) was fabricated by engraving the specific pathway onto a Viton® sheet using a plotter machine. Viton®, which was also used as the material for the cell's sealing frame, was selected for its chemical inertness. The flow field features a serpentine pathway with four turns, measuring 2.5 cm in length and 0.3 cm in width, and an overall thickness of 0.1 cm. FF also includes an inlet at the top and an outlet at the bottom to facilitate the eventual liquid formation and/or flooding of the electrolyte. The detailed layout of the pathway is illustrated in Figure S12.

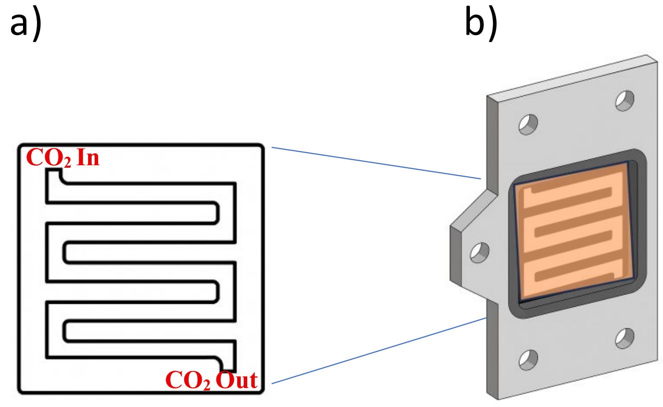


**Figure S12**: Flow field schematic illustration of serpentine-type pathway (a) and illustration of flow field-integrated WE (orange colour represents GDE or MEA) (b)

The as-prepared serpentine flow field was integrated into the titanium current collector (WE frame) window (with an area of 10 cm²) and sealed using insulating tape, such as Kapton®, to prevent leakage and ensure proper electrical isolation. The GDE (represented in Figure S9b in orange) for the L-FC configuration is positioned on top of the FF, with the catalyst layer facing the catholyte (Figures S13a and b). Similarly, for the G-FC configuration, the MEA faces the anolyte (Figure 1b in the manuscript). The backside of the GDE (or MEA), corresponding to the macroporous layer, is in direct contact with the FF, through which $CO_2$ is supplied and forced to follow the serpentine channel pathway. $CO_2$ diffuses through the macroporous layer into the microporous layer of the GDE, ultimately reaching the catalyst layer. The gaseous reaction products (e.g., $C_2H_4$, CO), along with unreacted $CO_2$ and $H_2$, then back-diffuse through the same layers and exit the cell along the same path.

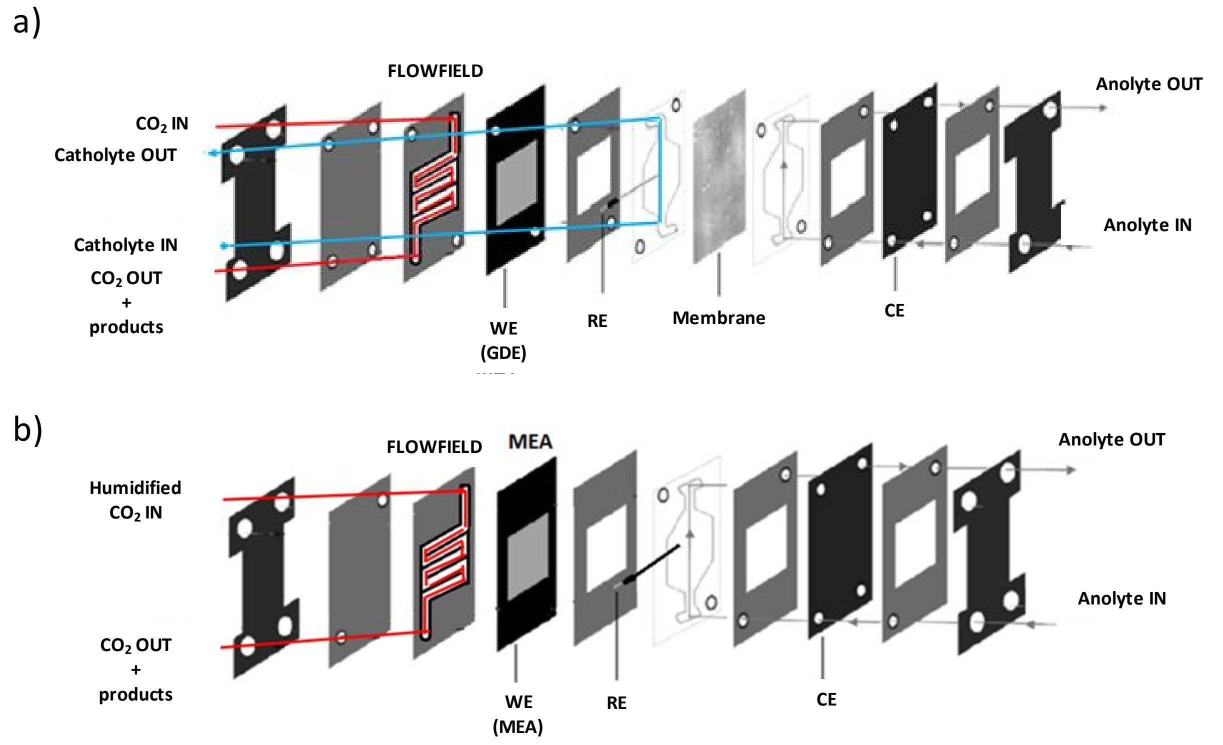


**Figure S13**: Schematic diagrams of the flow field-integrated electrocatalytic cell setups: L-FC (a) and G-FC (b)

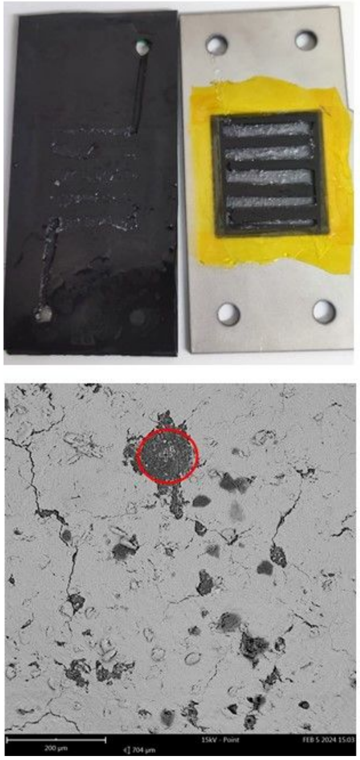

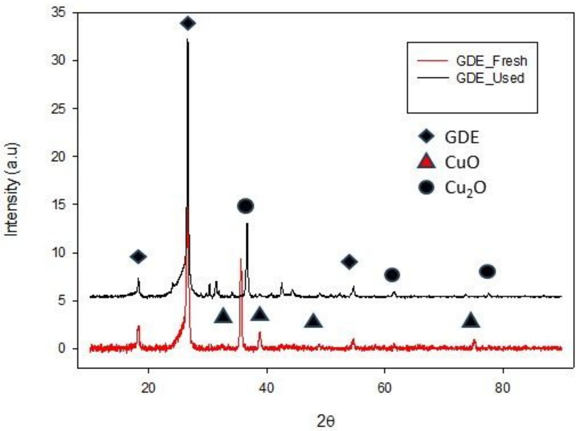


| EDX | Cu | C | O | K | F |
|---|---|---|---|---|---|
| Atomic Concentration (%) | 0.5 | 32.1 | 24.4 | 17.4 | 21.3 |

**Figure S14:** Post-use characterisation of CuONS-based GDE in G-FC setup using Nafion membrane

## Additional References


[1] Zhang, L.; Li, X.; Chen, L.; Zhai, C.; Tao, H. Honeycomb-like CuO@C for electroreduction of carbon dioxide to ethylene. *Journal of Colloid and Interface Science* **2023**, *640*, 783-790. DOI: https://doi.org/10.1016/j.jcis.2023.02.145.

[2] Li, P.; Bi, J.; Liu, J.; Wang, Y.; Kang, X.; Sun, X.; Zhang, J.; Liu, Z.; Zhu, Q.; Han, B. p–d Orbital Hybridization Induced by p-Block Metal-Doped Cu Promotes the Formation of C2+ Products in Ampere-Level CO2 Electroreduction. *Journal of the American Chemical Society* **2023**, *145* (8), 4675-4682. DOI: 10.1021/jacs.2c12743.

[3] Mallik, M.; Monia, S.; Gupta, M.; Ghosh, A.; Toppo, M. P.; Roy, H. Synthesis and characterization of Cu2O nanoparticles. *Journal of Alloys and Compounds* **2020**, *829*, 154623. DOI: https://doi.org/10.1016/j.jallcom.2020.154623.

[4] Duong, T.-H.; Kim, H.-C. A high productivity and speedy synthesis process for copper nanowires via an ethylenediamine-mediated method. *International Nano Letters* **2017**, *7* (2), 165-169. DOI: 10.1007/s40089-017-0204-4.

[5] Jalal, A.; Zhao, Y.; Uzun, A. Pyrolysis Temperature Tunes the Catalytic Properties of CuBTC-Derived Carbon-Embedded Copper Catalysts for Partial Hydrogenation. *Industrial & Engineering Chemistry Research* **2022**, *61* (5), 2068-2080. DOI: 10.1021/acs.iecr.1c04741.

[6] Yin, Z.; Yu, J.; Xie, Z.; Yu, S.-W.; Zhang, L.; Akauola, T.; Chen, J. G.; Huang, W.; Qi, L.; Zhang, S. Hybrid Catalyst Coupling Single-Atom Ni and Nanoscale Cu for Efficient CO2 Electroreduction to Ethylene. *Journal of the American Chemical Society* **2022**, *144* (45), 20931-20938. DOI: 10.1021/jacs.2c09773.

[7] M. Ignat, C. J. Van Oers, J. Vernimmen, M. Mertens, S. Potgieter-Vermaak, V. Meynen, E. Popovici and P. Cool, Textural property tuning of ordered mesoporous carbon obtained by glycerol conversion using SBA-15 silica as template, *Carbon* **2010**, 48, 1609-1618. https://doi.org/10.1016/j.carbon.2009.12.062.
